# Epigenetic priming in chronic liver disease impacts the transcriptional and genetic landscapes of hepatocellular carcinoma

**DOI:** 10.1002/1878-0261.13154

**Published:** 2021-12-29

**Authors:** John Gallon, Mairene Coto‐Llerena, Caner Ercan, Gaia Bianco, Viola Paradiso, Sandro Nuciforo, Stephanie Taha‐Melitz, Marie‐Anne Meier, Tujana Boldanova, Sofía Pérez‐del‐Pulgar, Sergio Rodríguez‐Tajes, Markus von Flüe, Savas D. Soysal, Otto Kollmar, Josep M. Llovet, Augusto Villanueva, Luigi M. Terracciano, Markus H. Heim, Charlotte K. Y. Ng, Salvatore Piscuoglio

**Affiliations:** ^1^ Visceral Surgery and Precision Medicine Research Laboratory Department of Biomedicine University of Basel Switzerland; ^2^ Institute of Medical Genetics and Pathology University Hospital Basel Switzerland; ^3^ Hepatology Laboratory Department of Biomedicine University of Basel Switzerland; ^4^ Clarunis Department of Visceral Surgery University Centre for Gastrointestinal and Liver Diseases St. Clara Hospital and University Hospital Basel Switzerland; ^5^ Liver Unit Hospital Clinic IDIBAPS CIBERehd University of Barcelona Spain; ^6^ Translational Research in Hepatic Oncology Liver Unit IDIBAPS Hospital Clínic University of Barcelona Spain; ^7^ Liver Cancer Program Divisions of Liver Diseases and Hematology/Medical Oncology Department of Medicine Tisch Cancer Institute Icahn School of Medicine at Mount Sinai New York NY USA; ^8^ Department of Pathology Humanitas Clinical and Research Center IRCCS Milan Italy; ^9^ Department of Biomedical Sciences Humanitas University Milan Italy; ^10^ Department for BioMedical Research University of Bern Switzerland; ^11^ SIB Swiss Institute of Bioinformatics Lausanne Switzerland

**Keywords:** chronic liver disease, epigenetic priming, hepatocellular carcinoma, methylation

## Abstract

Hepatocellular carcinomas (HCCs) usually arise from chronic liver disease (CLD). Precancerous cells in chronically inflamed environments may be ‘epigenetically primed’, sensitising them to oncogenic transformation. We investigated whether epigenetic priming in CLD may affect HCC outcomes by influencing the genomic and transcriptomic landscapes of HCC. Analysis of DNA methylation arrays from 10 paired CLD‐HCC identified 339 shared dysregulated CpG sites and 18 shared differentially methylated regions compared with healthy livers. These regions were associated with dysregulated expression of genes with relevance in HCC, including ubiquitin D (*UBD*), cytochrome P450 family 2 subfamily C member 19 (*CYP2C19*) and *O*‐6‐methylguanine‐DNA methyltransferase (*MGMT*). Methylation changes were recapitulated in an independent cohort of nine paired CLD‐HCC. High CLD methylation score, defined using the 124 dysregulated CpGs in CLD and HCC in both cohorts, was associated with poor survival, increased somatic genetic alterations and *TP53* mutations in two independent HCC cohorts. Oncogenic transcriptional and methylation dysregulation is evident in CLD and compounded in HCC. Epigenetic priming in CLD sculpts the transcriptional landscape of HCC and creates an environment favouring the acquisition of genetic alterations, suggesting that the extent of epigenetic priming in CLD could influence disease outcome.

## Graphical abstract

Precancerous cells in chronically diseased livers (CLD) may be ‘epigenetically primed’, sensitising them to oncogenic transformation. We investigated whether epigenetic priming in CLD also affects hepatocellular carcinoma (HCC) outcome. We found this sculpts the HCC transcriptome, creating an environment favouring the acquisition of genetic alterations. Our DNA methylation‐based score shows the extent to which epigenetic priming occurs in CLD and influences HCC outcome.

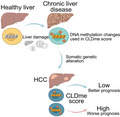

**Abbreviations**
CLD: chronic liver disease
CLDme: chronic liver disease methylation
DE: differentially expressed
DM: differential methylation
DMR: differentially methylated regions
EMT: epithelial–mesenchymal transition
HCC: hepatocellular carcinoma
NAFLD: nonalcoholic fatty liver disease
PC: principal component
SAM: *S*‐adenosylmethionine
SNVs: single nucleotide variants
VAF: variant allelic fraction

## 1 Introduction

Hepatocellular carcinoma (HCC) typically arises in the context of chronic inflammation and tissue necrosis [1]. Viral infections, excessive alcohol consumption, ingestion of aflatoxin B1 and nonalcoholic fatty liver disease (NAFLD) are all well‐defined causes of chronic liver disease (CLD) and risk factors for HCC development [2]. Regardless of the aetiology, hepatocarcinogenesis usually occurs as a multistep progression from the healthy liver to fibrosis, cirrhosis and ultimately HCC, a process that relies heavily on changes in the tissue microenvironment and the accumulation of epi/genetic alterations in the hepatocytes and stellate cells [3, 4, 5].

The concept of epigenetic priming has been proposed in other cancers emerging from chronic health conditions or environmental factors, such as obesity in colon cancer or cigarette smoke in lung cancer [6, 7]. In this model, precancerous cells assume a new, epigenetically defined identity, which sensitises them to oncogenic transformation. Similar to these cancers, HCC arises from a background of chronic disease. Indeed, epigenetic dysregulation was initially reported in CLD, with hypermethylation of the promoters of tumour suppressors such as *RASSF1A*, *APC* and *CDKN2A* [8, 9, 10]. These studies demonstrated that select epigenetic alterations that exist in HCC are also present in CLD, suggesting that they may contribute to disease initiation and/or progression. Subsequently, DNA methylation changes in NAFLD have been associated with aberrant gene expression in nontumoral tissue, while genome‐wide analysis of methylation patterns has revealed the extent of epigenetic dysregulation in precancerous nodules [8, 11, 12]. The prognostic utility of DNA methylation patterns in HCC, following tumorigenesis, has also been demonstrated, and particular DNA methylation signatures have recently been linked to specific driver gene alterations [13, 14].

This literature points to critical roles for epigenetic changes acquired during CLD in the initial emergence of HCC, and for those acquired during HCC on disease progression. However, the impact on the transcriptional and genetic landscapes of HCC, and prognostic utility of genome‐wide DNA methylation changes acquired specifically during CLD remains unexplored. Here, we identify genome‐wide DNA methylation changes acquired in nontumoral CLD tissue, associated with distinct transcriptional and genetic landscapes in tumour samples. Using the results obtained from these analyses, we developed a score that may have prognostic value in HCC.

## 2 Materials and methods

### 2.1 Patients and samples

For the discovery cohort, 10 patients with HCC were diagnosed at the University Hospital Basel and were prospectively recruited for this study after written informed consent. HCC biopsies, concomitant CLD biopsies and peripheral blood leucocytes were collected from the HCC patients (Fig. S1 and Table S1A).

From each patient undergoing a diagnostic liver biopsy, two ultrasound‐guided core needle biopsies of the primary tumour and two biopsies from the CLD tissue and whole blood were collected at diagnosis at the same time. Of the two biopsies taken from the primary tumour and from CLD tissue, one was processed and embedded in paraffin for clinical purposes and the other one was snap‐frozen and stored at −80 °C for research purposes. Ten millilitres of whole blood was collected and processed immediately for the isolation of peripheral blood leucocytes (‘buffy coat’). All biopsies were histologically characterised by two hepatopathologists (CE and LMT) to confirm the initial diagnosis of HCC [15]. The study was performed in accordance with the Declaration of Helsinki, and the approval for the use of these samples was granted by the ethics committee (Protocol Number EKNZ 2014‐099).

For the validation cohort, nine patients with HCC and concomitant CLD diagnosed at the Hospital Clinic, Barcelona or Mount Sinai, New York, were prospectively recruited after written informed consent [Protocol Number 2010/5896 (IRB Hospital Clinic, Barcelona), Fig. S1 and Table S1A].

As controls for methylation array profiling, healthy livers from two patients with colorectal cancer metastatic to the liver (University Hospital Basel, Protocol Number EKNZ 2014‐099) and histologically normal tissues from 10 patients undergoing hepatic resection due to non‐cancer‐related diseases [Protocol Number 2010/5896 (IRB Hospital Clinic, Barcelona)] were used. As controls for transcriptomic analysis, liver biopsies with normal histology obtained from 15 patients without HCC and with normal liver values were used (University Hospital Basel, Protocol Number EKNZ 2014‐099, Fig. S1).

For all patients in the discovery and validation cohorts, the clinical staging was determined according to the Barcelona Clinic Liver Cancer staging system [16]. Sex and age of the patients, clinical diagnosis, and underlying liver disease (hepatitis B/C infection, alcoholic liver disease, NAFLD) were retrieved from clinical files (Table S1A).

The samples encompassed the diverse backgrounds of HCC; of the 10 patients, 5 were diagnosed with alcohol‐related HCC, 3 with HBV/HCV‐related HCC and 2 with NAFLD‐related HCC (Table S1A). Our discovery cohort of 10 patients largely consisted of early‐stage tumours (70% BCLC stages 0‐A) with nonmultinodular HCC (70% < 2 nodules). Using the data generated from these samples, we investigated how transcriptional changes might drive disease progression.

### 2.2 Nucleic acid extraction

Genomic DNA and total RNA from biopsies from the discovery cohort were extracted using the ZR‐Duet DNA and RNA MiniPrep Plus Kit (Zymo Research, Freiburg im Breisgau, Germany) following the manufacturer’s instructions. Prior to extraction, biopsies were crushed in liquid nitrogen to facilitate lysis. Extracted DNA was quantified using the Qubit Fluorometer (Invitrogen, Waltham, MA, USA) [17]. DNA from peripheral blood leucocytes (‘buffy coat’) was extracted using the DNeasy Blood and Tissue Kit (Qiagen, Hilden, Germany) according to the manufacturer’s instructions. For the validation cohort, DNA was extracted using the ChargeSwitch Genomic DNA Mini Tissue Kit (Invitrogen) following the manufacturer’s instructions [8].

### 2.3 Exome sequencing and analysis

Whole‐exome capture was performed using the SureSelect XT Clinical Research Exome (Agilent, Santa Clara, CA, USA) platform according to the manufacturer’s guidelines (Fig. S1). Sequencing was performed on an Illumina HiSeq 2500 at the Genomics Facility Basel according to the manufacturer’s guidelines. Paired‐end 101‐bp reads were generated. Reads obtained were aligned to the reference human genome GRCh37 using Burrows‐Wheeler Aligner (bwa, v0.7.12) [18]. Local realignment, duplicate removal and base quality adjustment were performed using the Genome Analysis Toolkit (gatk, v3.6) and picard (http://broadinstitute.github.io/picard/) [19]. Somatic single nucleotide variants (SNVs) and small insertions and deletions (indels) were detected using mutect (v1.1.4) and strelka (v1.0.15), respectively [20, 21]. We filtered out SNVs and indels outside of the target regions, those with a variant allelic fraction (VAF) of < 1% and/or those supported by < 3 reads. We also excluded variants for which the tumour VAF was < 5 times that of the paired nontumour VAF. We further excluded variants identified in at least two of a panel of 123 nontumour samples, including the nontumour samples included in the current study, captured and sequenced using the same protocols using the artefact detection mode of mutect implemented in gatk. To account for the presence of somatic mutations that may be present below the limit of sensitivity of somatic mutation callers, we used gatk Unified Genotyper to interrogate the positions of all unique mutations in all samples from a given patient to define the presence of additional mutations. Variants identified by this genotyping step supported by a minimum of two reads are annotated as ‘Genotyped’. Hot spot missense mutations were annotated using the published resources [22, 23].

Allele‐specific copy‐number alterations were identified using facets (v0.5.6), which performs joint segmentation of the total and allelic copy ratios and infers purity, ploidy and allele‐specific copy‐number states [24]. Copy‐number states were collapsed to the gene level using the median values to coding gene resolution based on all coding genes retrieved from the Ensembl (release GRCh37.p13). Genes with total copy number greater than gene‐level median ploidy were considered gains: greater than ploidy + 4, amplifications; less than ploidy, losses; and total copy number of 0, homozygous deletions. Somatic mutations associated with the loss of the wild‐type allele [i.e., loss of heterozygosity (LOH)] were identified as those for which the lesser (minor) copy‐number state at the locus was 0. All mutations on chromosome X in male patients were considered to be associated with LOH [25].

### 2.4 RNA sequencing and analysis

Two hundred nanogram total RNA was used for RNA‐seq library prep with the TruSeq Stranded Total RNA Library Prep Kit with Ribo‐Zero Gold (Illumina, San Diego, CA, USA) according to manufacturer’s specifications (Fig. S1). Sequencing was performed on an Illumina HiSeq 2500 using v4 SBS chemistry at the Genomics Facility Basel according to the manufacturer’s guidelines. Sequence reads were aligned to the human reference genome GRCh37 by star using the two‐pass approach [26]. Transcript quantification was performed using rsem [27]. Genes without > 10 counts in at least two samples were discarded. Counts were normalised using the median of ratios method from the deseq2 package in R version 3.6.1 [28].

Comparisons of the intragroup variation, defined as the within‐group pairwise Euclidean distance based on their principal components (PCs), were performed using Wilcoxon’s tests. Differential expression analysis was performed using the Wald test in deseq2 [29]. Genes with |logFC| > 1.5 and FDR < 0.05 were considered differentially expressed (DE). Gene set enrichment analysis was performed using the fgsea package using Hallmark gene sets, with genes ranked based on the *t* statistic from deseq2 [29, 30].

### 2.5 Methylation profiling and analysis

Methylation profiling was performed using Infinium^®^ MethylationEPIC BeadChip and Infinium^®^ HumanMethylation450 BeadChip (Illumina) on the discovery and validation cohorts, respectively (Fig. S1). After whole‐genome amplification and enzymatic fragmentation, the samples were hybridised to the BeadChip and scanning was conducted with the Illumina iScan. Idat files were exported and analysed using the minfi package in R [31]. All arrays were reduced to probes present on both the HumanMethylation450 and MethylationEPIC BeadChips, as 10 of 12 normal samples, those from Barcelona, were analysed on the HumanMethylation450 BeadChip. Probes associated with SNPs, on the sex chromosomes, or with a detection *P* value > 0.01 in any sample were removed prior to analysis. Data were normalised using the Noob algorithm from the minfi package [31]. Probes were annotated using the IlluminaHumanMethylation450kanno package in bioconductor.

Principal component analysis was performed using the top 500 most variable CpG sites. Comparisons of the intragroup variation, defined as the within‐group pairwise Euclidean distance based on their PCs, were performed using Wilcoxon’s tests. Comparisons of the intergroup variation, as measured by pairwise Euclidean distance based on their PCs between samples of different groups, were performed using Wilcoxon’s tests. Probe‐level differential methylation analysis was performed for 42 925 CpG sites using limma. Probes with |logFC| > 1.5 and FDR < 0.05 were considered differentially methylated (DM). Differentially methylated regions (DMRs) were called using DMRcate using the parameters ‘lambda=500, C=5’. [32, 33, 34] DMRs with mean change in *B* value > |15%| and FDR < 0.05 were considered differentially methylated. DMRs were annotated using the annotateTranscripts function from the bumphunter and the TxDb.Hsapiens.UCSC.hg19.knownGene packages from bioconductor [35].

To assess the relationship between DMRs and methyl‐binding domain proteins and repressive histone modifications, we downloaded ENCODE ChIP‐seq data for ZBTB38, ZBTB4 and Histone 3 Lysine 27 trimethylation [36], and intersected these with the DMRs using bedtools [37].

### 2.6 Downloading and annotation of the TCGA cohort

DNA methylation, gene expression, mutation and survival data for 430 HCC samples were downloaded from TCGA using the TCGAbiolinks package in R on 28 July 2020 [38, 39]. Copy‐number alteration data were downloaded from TCGA Firehose [40]. Assessment of the presence or absence of cholestasis, Mallory bodies, tumour‐infiltrating lymphocytes, vessel infiltration and necrotic areas was performed as previously described [41]. TCGA samples were reduced to the 368 for which complete DNA methylation data, survival data and histological annotation were available.

### 2.7 Development of CLD DNA methylation (CLDme) prognostic score

One hundred and twenty‐four probes were DM in CLD and HCC in both the discovery and validation cohorts; after removing 15 of 124 probes with NA values in the TCGA data set, an elastic net Cox regression model was built using the remaining 109 probes and overall survival as the response variable. Elastic net regression is a regularisation method that balances the trade‐off between bias and variance using L1 and L2 regularisation parameters [42]. These are combined into a single parameter, lambda, in the implementation of elastic net regression in the glmnet r package [43]. The optimal value for lambda was selected using the training set and 10‐fold cross‐validation using the cv.glmnet function from the glmnet package [43]. The model was built on a training set consisting of a randomly selected 70% (*n* = 257) of the 368 TCGA HCC samples. A fixed seed was used in order to ensure reproducibility. The remaining 30% (*n* = 111) samples were reserved for testing. Samples were classified as CLDme score high or low based on the median score of samples in the TCGA training set after defining the optimal value for lambda. Differences in survival between CLDme high/low groups were compared using the log‐rank test, adjusted for disease history and stage (the only factors significantly associated with survival).

### 2.8 Analysis of TCGA samples stratified by CLDme score

To compare the gene expression profiles between CLDme high and low samples, differential gene expression analysis was performed using 362 TCGA samples for which DNA methylation, and transcriptomic and clinical information were available. Differential gene expression analysis was performed using the Wald test in deseq2 [29]. Comparisons of numbers of mutations, and copy‐number alterations between CLDme high and low samples were carried out using Wilcoxon’s tests on the 306 and 364 TCGA samples for which clinical, DNA methylation and mutation/copy‐number data were available, respectively. Comparison of lymphocyte invasion between CLDme high/low groups was carried out using the histological annotation as described previously. ImmuneScores for each TCGA sample were downloaded from https://xcell.ucsf.edu/ and compared between CLDme high/low groups [44].

### 2.9 Immunohistochemistry

Immunohistochemical staining was performed on a Benchmark immunohistochemistry staining system (Bond; Leica, Wetzlar, Germany) with BOND polymer refine detection solution for DAB, using anti‐MGMT (1 : 800, abcam, Cambridge, UK, ab39253) primary antibody as substrate as previously described [45]. Images were acquired using an Olympus BX46 microscope (Shinjuku City, Tokyo, Japan) as previously described. MGMT immunoreactivity was scored semi‐quantitatively by multiplying the proportion of MGMT positive cells (%) and the staining intensity (0 = none; 1 = weak; 2 = intermediate; and 3 = strong). Statistical comparison was performed using paired Wilcoxon test.

## 3 Results

### 3.1 Transcriptional alterations present in HCC are detectable in CLD tissue

**Fig. 1:**
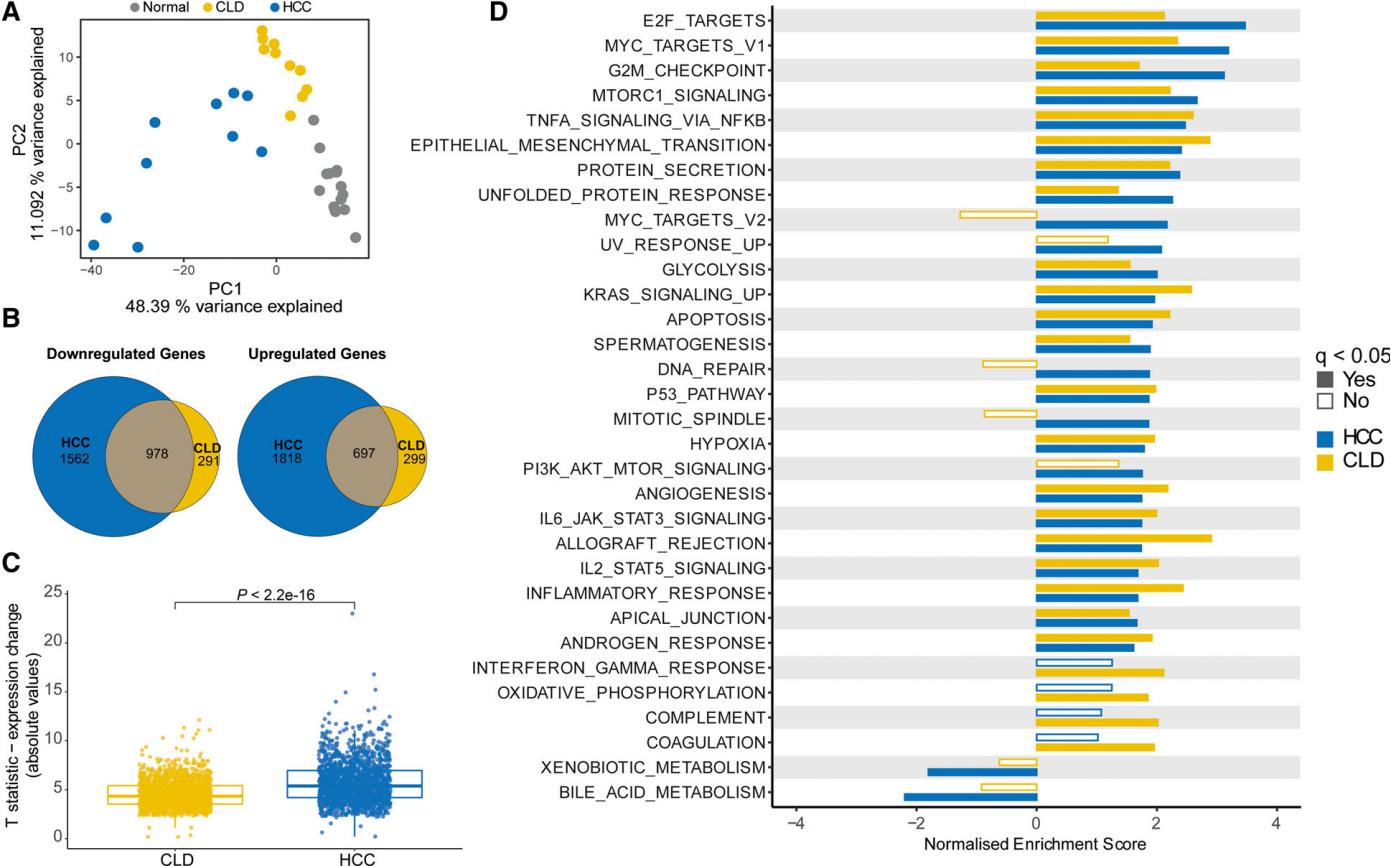
Oncogenic transcriptional alterations in CLD are compounded in HCC. (A) PC analysis of 15 healthy liver and 10 paired CLD and HCC samples. (B) Venn diagrams of down‐ and upregulated genes in CLD and HCC compared with normal (|log_2_FC| > 1.5 and *q* < 0.05). (C) *T* statistics (absolute values) of 1675 DE genes in both CLD and HCC compared to normal livers. DE: |log2FC| > 1.5, *q < *0.05. *P* computed from paired Wilcoxon tests. (D) Hallmark pathways with significant enrichment in CLD, HCC or both from gene set enrichment analysis are shown (GSEA; *P* < 0.05). CLD: chronic liver disease; HCC: Hepatocellular carcinoma; DE: differentially expressed.

To identify transcriptional alterations in diseased liver tissues that progressed to HCC, we performed RNA sequencing on needle biopsies from 10 HCC tissue and matched adjacent CLD tissue, along with 15 healthy liver samples against which CLD and HCC transcriptional profiles were compared (Fig. S1). Unsupervised analysis of gene expression data showed that normal and HCC samples form distinct clusters (Fig. 1A), with CLD tissues clustering closer to the normal tissues than HCCs. This was reflected in unsupervised consensus clustering, which showed normal and HCC clustering separately, with CLD tissues split between these two clusters (Fig. S2). Differential gene expression analysis detected a significant overlap between transcriptional alterations in CLD and HCC when compared to normal samples. Nine hundred and seventy‐eight of 1269 (77.1%) and 697 of 996 (70.0%) genes down‐ and upregulated, respectively, in CLD were also DE in HCC (both *P* < 0.0001, hypergeometric tests, Fig. 1B, Table S1B–D, Fig. S3). HCCs, however, acquired a further 1562 and 1818 genes down‐ and upregulated, respectively. Furthermore, the change in expression of the 1675 genes showing DE in both CLD and HCC was significantly amplified in HCC compared with CLD (*P* < 2.22e^−16^, paired Wilcoxon test, Fig. 1C).

Pathway analysis of the dysregulated genes show upregulation of epithelial‐to‐mesenchymal transition (EMT)‐related genes in CLD and HCC (Fig. 1D), consistent with the tissue regeneration and fibrogenic processes occurring during CLD [30]. Interestingly, cancer‐related pathways, such as cell cycle (MYC targets V1) and MTORC1 signalling, were also upregulated in both HCC and CLD samples, suggesting that these pathways may already be transcriptionally dysregulated in the precancerous lesion. The magnitude of upregulation of these pathways was greater in the HCCs than in the CLDs, highlighting the progressive nature of these changes. By contrast, we also found upregulation of DNA repair and mitotic spindle pathways and downregulation of the xenobiotic and bile acid metabolism in HCC samples, but not the CLD samples (Fig. 1D). Conversely, we found significant alteration of the complement and interferon gamma response pathways in the CLD samples but not the HCC.

To determine whether the transcriptional alterations observed in CLD were driven by somatic genetic alterations, we performed whole‐exome sequencing on the matched CLD and HCC samples (Fig. S1). We detected at least one somatic mutation in the most commonly mutated genes in HCC [46] and substantial copy‐number alterations in 9 of 10 HCCs (Fig. S4, Table S1E,F). However, except for one low‐confidence mutation in *APOB* in the CLD from patient 6, we found no evidence for shared mutations in the commonly mutated genes or copy‐number alterations between CLD and HCC samples from the same patient.

Together, these data demonstrate the significant accumulation of cancer‐associated transcriptional changes in CLD, which are compounded in HCC, and suggest that the aberrant transcriptional landscape of HCC may start developing during CLD independent of genetic alterations.

### 3.2 DNA methylation alterations in HCC are detectable in CLD

Given that cancer‐associated transcriptional changes in CLD do not appear to be underpinned by genomic changes frequently observed in HCC, we asked whether epigenetic alterations may help explain the transition towards HCC. In support of this hypothesis, we found progressive loss of expression of *MAT1A* (CLD *q* = 0.02, Log2FC = −0.80, HCC *q* = 1.43e^−11^, Log2FC = −2.14; Table S1B,C), which catalyses synthesis of the universal methyl donor *S*‐adenosylmethionine (SAM) as previously reported in cirrhotic livers [47]. As the loss of SAM availability suggests the potential for epigenetic reprogramming, we subjected the same 10 pairs of CLD and HCC, and 12 normal liver samples to methylation profiling (Fig. S1).

**Fig. 2:**
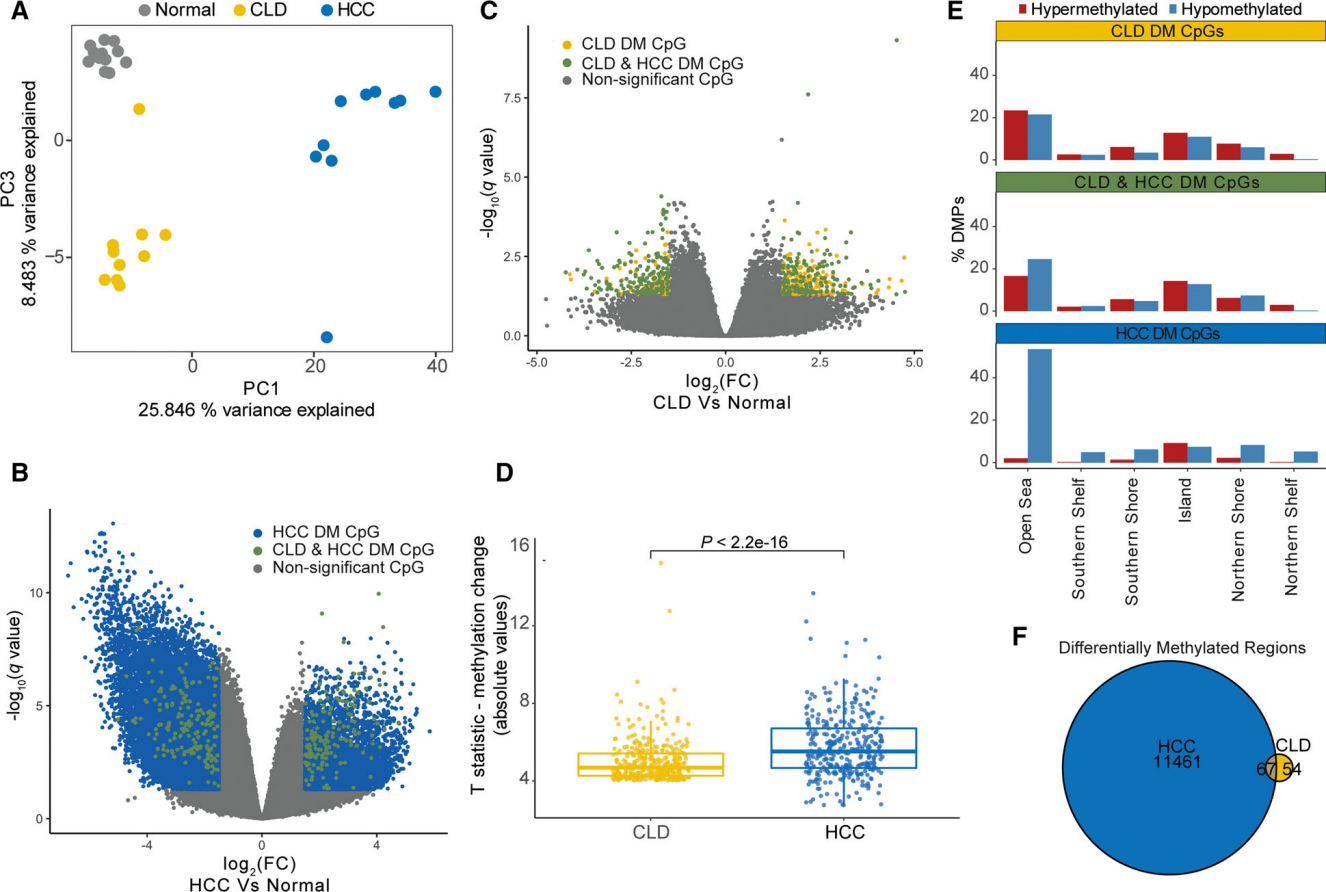
DNA methylation alterations in HCC are detectable in CLD. (A) PC analysis of 12 healthy liver samples, and 10 paired CLD and HCC samples, showing PC1 and PC3. (B, C) Differential methylation analysis [−log_10_(*q*) against log_2_ fold change (*M* value)] comparing CLD (B) and HCC (C) with healthy livers. Significant CpG sites (|log_2_ fold change| > 1.5, *q* < 0.05) are coloured according to the legend. (D) *T* statistics (absolute values) of 339 differentially methylated CpG sites in both CLD and HCC compared with normal livers. DM: |log2FC| > 1.5, *q < *0.05. *P* computed from paired Wilcoxon test. (E) Distribution of differentially methylated CpG sites (DMPs) according to their genomic features, detected in 10 CLD and 10 HCC compared with 12 normal livers. (F) Venn diagram showing intersection of DMRs called in CLD and HCC samples compared with normal livers. PC: Principal component; CLD: chronic liver disease; HCC: Hepatocellular carcinoma; DM: differentially methylated; DMP: differentially methylated probes; DMR: differentially methylated region.

Principal component analysis of the methylation profiles reflected the findings from the transcriptional analysis; CLD/normal livers were separated from HCCs by PC1, but CLDs were separated from normal livers by PC3 (Fig. 2A, Fig. S5A), reflecting a recent study showing a gradient of methylation changes spanning the progression from health liver to HCC [48]. We identified 54 888 differential methylated (DM, |log2FC| > 1.5, *q* < 0.05) CpG sites in the HCC samples compared with normal tissue, the majority of which (46 669, 85%) were hypomethylated (Fig. 2B, Table S1G), consistent with the phenomenon of genome‐wide hypomethylation in cancer cells [49, 50]. Differential methylation was observed at CpGs associated with *P14* and *RASSF1A*, previously shown to be aberrantly methylated in HCC (Fig. S5B) [51, 52, 53]. In the CLD samples, we detected 586 DM CpGs compared with normal liver (Fig. 2C, Table S1H). Of these, 339 CpGs, associated with 222 genes, were also DM in the HCC samples, representing a highly significant overlap (*P* < 0.0001, hypergeometric test, Fig. 2C, Fig. S6, Table S1I). Importantly, as with the genes that were DE in both CLD and HCC, the 339 CpG sites that were differentially methylated in both CLD and HCC compared with normal showed significantly larger methylation changes in HCC than in CLD (*P* < 2.22e^−16^, paired Wilcoxon’s test; Fig. 2D). Compared with HCC, a greater proportion of the methylation changes observed in CLD had the potential to regulate gene expression. In HCC samples, 53.5% DM CpG sites were hypomethylated and in Open Sea regions (> 4 kb from a CpG island), compared with 21.5% in CLD samples. On the contrary, DM CpGs in CLD samples were enriched in CpG island and shore regions (< 2 kb from a CpG island) compared with HCC (*P* = 0.016, OR = 1.36, Fisher’s exact test; Fig. 2E), which could suggest that methylation alterations in CLD have proportionally greater effect on transcriptional regulation than those in HCC.

Given that differentially methylated regions (regions of adjacent CpG sites showing significantly altered methylation (DMRs)) have been shown to be more strongly linked to gene expression than methylation changes at single CpG sites [54], we further identified DMRs (mean change in *B* value >|0.15|, *q* < 0.05) in CLD and HCCs [32]. As with the probe‐level analysis, we detected substantially more DMRs in the HCC samples than in the CLD samples, compared with the normal (11 582 and 121, respectively). Intersecting these regions identified 67 DMRs, containing 262 CpGs, showing altered methylation in both CLD and HCC samples (Fig. 2F).

Our data demonstrate the extent of epigenetic changes in CLD and that many of those changes are amplified in HCC. As genetic alterations typically observed in HCC were not detected in CLD, while HCC‐associated methylation changes were evident, this suggests the aberrant methylome of HCC may, in part, have emerged before tumorigenesis.

### 3.3 DNA methylation changes in CLD sculpt the transcriptional landscape of HCC

**Fig. 3:**
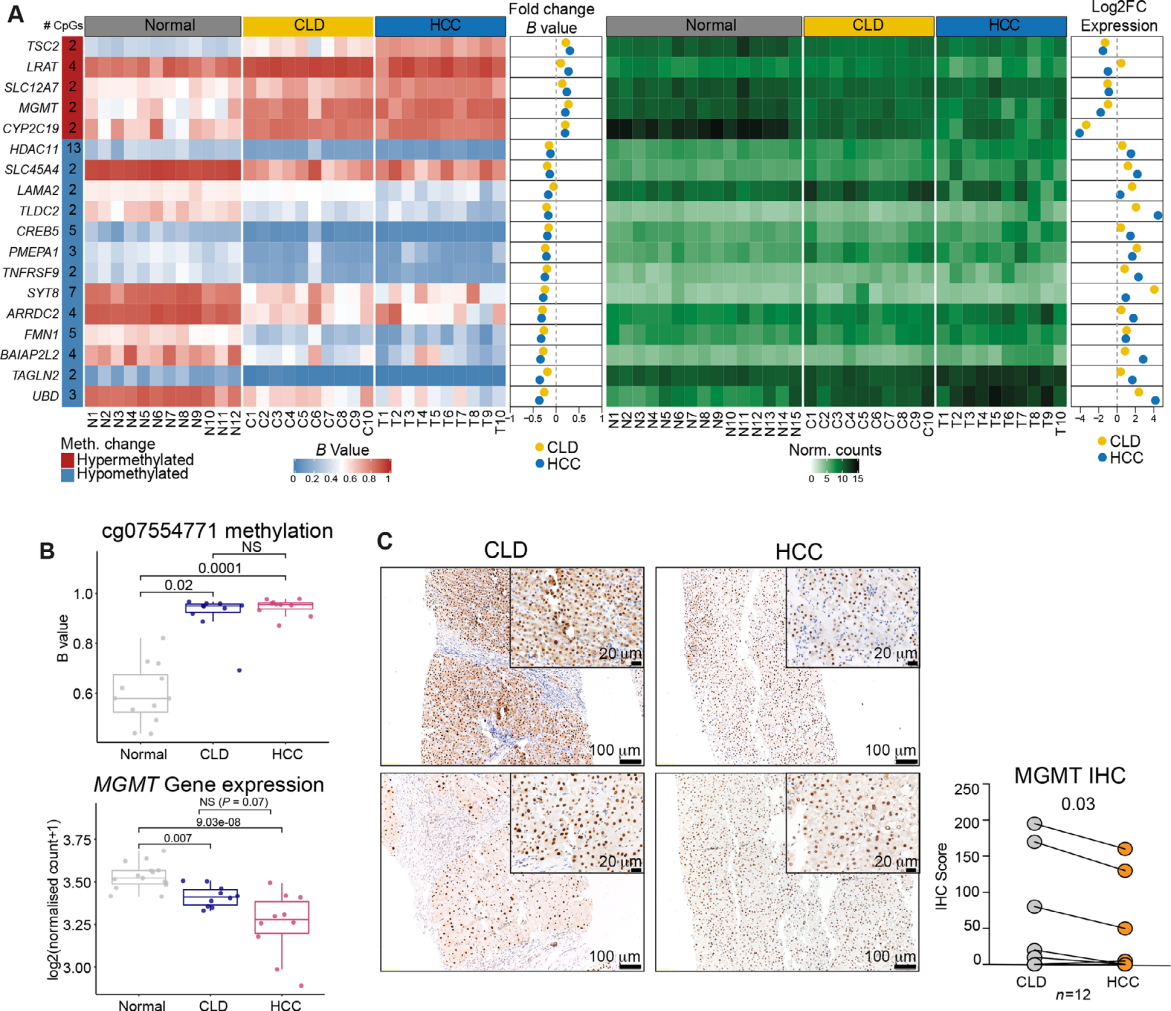
Gene expression and DNA methylation changes define the transition from CLD to HCC. (A) Heatmaps of methylation and gene expression at DE genes associated with DMRs (|mean change in methylation *B* value| > 0.15 and FDR < 0.05). Dot plots showing mean change in *B* value (methylation) and log_2_ fold change (gene expression) between CLD (*n* = 10) and normal (*n* = 12) and HCC (*n* = 10) and normal (*n* = 12). (B) cg07554771 methylation and *MGMT* expression from 10 paired CLD (*n* = 10) and HCC samples (*n* = 10). *P* values computed from limma (methylation) and deseq2 (gene expression). (C) Representative immunohistochemistry images from two paired CLD and HCC biopsies stained with anti‐MGMT antibody and MGMT protein expression IHC scores from 12 paired CLD and HCC samples (paired Wilcoxon’s test). Scale bar 20 and 100 µm. DMR: differentially methylated region; CLD: chronic liver disease; HCC: Hepatocellular carcinoma.

To determine how the DNA methylation changes observed in CLD and HCC shape their transcriptional profiles, we interrogated the 67 DMRs to search for those associated with DE genes (|log2FC| > 1.5, *q* < 0.05). We therefore removed candidate DMRs for which we did not have gene expression data, those that could not be associated with a gene promoter, that is annotated as ‘downstream’, and those whose change in methylation was not reflected in a significant change in gene expression, in the expected direction. This filtering left 18 regions differentially methylated in both CLDs and HCCs associated with DE genes (Fig. 3A and Table S1J,K). The genes affected by the epigenetic priming occurring in CLD included hypermethylated regions associated with the cytochrome P450 family gene *CYP2C19* and tuberous sclerosis complex 2 (*TSC2*), both downregulated in the CLD and HCC samples and reported to be lost in HCC with implications for prognosis [55, 56]. As an exploratory analysis to further demonstrate the relevance of these regions in the epigenetic regulation of gene expression, we found methyl‐binding domain protein (ZBTB4 and ZBTB38) and H3K27me^3^ peaks from a previously published study overlapped with the DMRs associated with *HDAC11*, *SYT8* and *TLDC2* [36], suggesting MBD proteins may interact with the identified DMRs (Fig. S7). We also identified a hypermethylated DMR within intron 3 of *MGMT*, containing the CpG site cg07554771 (CLD log2FC = 2.89, *q* = 0.02, HCC log2FC = 3.25, *q* = 0.0002; Fig. 3B, top), hypermethylation of which is correlated with *MGMT* repression in NAFLD and HCC [11]. Furthermore, an additional DMR was detected in the HCC samples, containing the CpG site cg00639517, hypermethylation of which is also correlated with loss of *MGMT* expression (Fig. S8) [11]. The hypermethylation of *MGMT* was concomitant with a loss of *MGMT* expression in CLD and HCC (CLD log2FC = −0.99, *q* = 0.007, HCC log2FC = −1.80, *q* = 9.04e^−8^; Fig. 3B, bottom). Corroborating the progressive loss of *MGMT* expression in HCC progression, immunohistochemical analysis of MGMT in an independent set of 12 matched CLD and HCC samples showed significant reduction in MGMT expression in HCC compared with matched CLD samples (*P* = 0.03, Wilcoxon’s test, Fig. 3C).

While studies on DNA methylation in CLD progression have mainly focussed on hypermethylation and silencing of tumour suppressor genes, 13 of the 18 identified DMRs showed hypomethylation and upregulation in the CLD and HCC samples compared with normal liver (Fig. 3A). These included the promoters of *UBD* (*FAT10*), a ubiquitin‐like modifier, and the calponin *TAGLN2*, both implicated in the progression of HCC, and *BAIAP2L2* coding for insulin receptor tyrosine kinase substrate, associated with actin remodelling and promoting HCC proliferation [57, 58, 59].

With the changes in DMRs reflected in gene expression changes in CLD and HCC, our findings demonstrate the potential for epigenetic priming in CLD, not only to influence tumorigenesis as has been extensively reported but also to sculpt the transcriptional landscape of the subsequent HCC.

### 3.4 CLD‐associated DNA methylation changes distinguish CLD and HCC from normal livers across cohorts

**Fig. 4:**
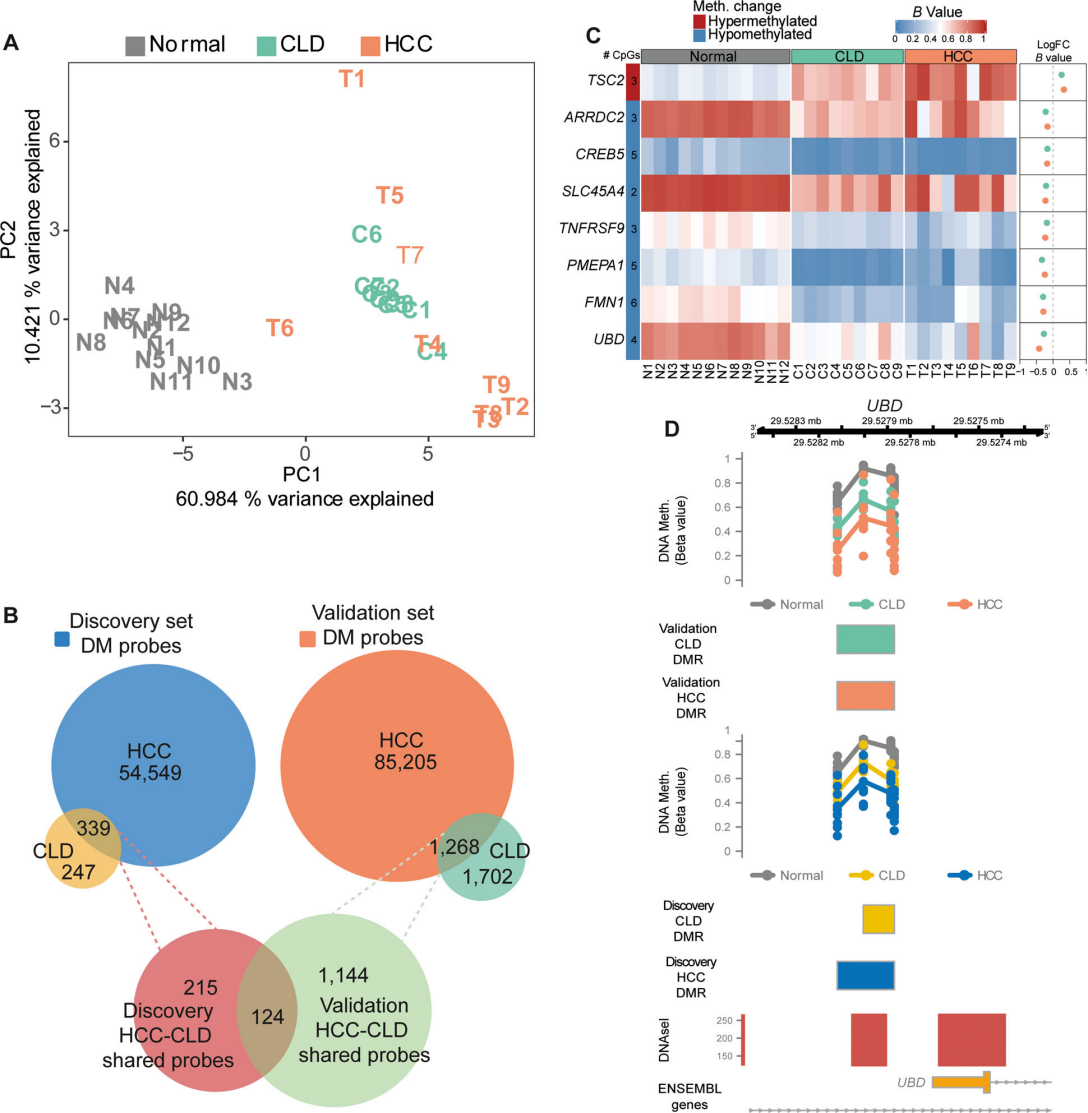
DNA methylation alterations in CLD and HCC are conserved across cohorts. (A) PC analysis of 12 healthy liver samples, and nine paired CLD and HCC samples from validation cohort, based on *B* values from the 51 CpG sites in the 18 DMRs associated with DE genes in the discovery cohort. (B) Venn diagrams showing overlap between DM CpG sites in CLD and HCC in the discovery and validation cohorts, and the overlap between CLD‐HCC DM CpG sites across cohorts. (C) Heatmap of methylation across normal, CLD and HCC samples at 8 DE genes associated with DMRs in HCC and CLD samples across both cohorts. Dot plot on right shows mean difference in *B* values between CLD and normal and HCC and normal. (D) Track plots showing *B* values at two CLD‐HCC overlapping DMRs in *UBD* in validation and discovery cohorts. DNAse hypersensitivity sites are denoted in the bottom tracks with ENSEMBL gene annotation. CLD: chronic liver disease; HCC: Hepatocellular carcinoma; DMR: differentially methylated region; DE: differentially expressed; DM: differentially methylated; DMR: differentially methylated region.

To rule out the possibility that the DNA methylation changes we detected in CLD were cohort‐specific, we analysed the DNA methylation data from an independent validation cohort of nine pairs of CLD and HCC samples (Fig. S1). PC analysis of the validation cohort using the *B* values of the 51 CpG sites in the 18 DE gene‐associated DMRs identified in both CLD and HCC in the discovery cohort (Fig. 3A) separated the normal samples from the CLD and HCC samples (Fig. 4A).

Differential methylation analysis of the samples in the validation cohort identified 2970 and 86 473 DM CpG sites in the CLD and HCC, respectively (|log2FC| > 1.5, *q* < 0.05, compared with normal livers). As in the discovery cohort, the overlap of 1268 DM CpG sites in both CLD and HCC in the validation cohort was highly significant (*P* < 0.0001, hypergeometric test; Fig. 4B). These CpG sites included those identified in genes already reported in the discovery cohort such as in *MGMT* (Fig. S9). Importantly, the overlap between the set of shared DM CpG sites identified in both cohorts (124 CpG sites) was also highly significant (*P* < 0.0001, hypergeometric test; Fig. 4B). The consistency of the observed methylation changes was also conserved at the DMR level where 8 of 18 identified CLD‐HCC DMRs, associated with DE genes in the discovery cohort, were shared with the validation cohort (Fig. 4C). Figure 4D shows the change in methylation between normal, and CLD and HCC samples at a representative gene promoter, *UBD*, found to be upregulated in the discovery cohort, concomitant with loss of methylation at its promoter. This was also observed in the validation cohort.

Together, these data suggest that specific epigenetic changes, with the potential to influence gene expression, occur consistently in CLD and are maintained in HCC.

### 3.5 Epigenetic priming in CLD creates a permissive environment for the accumulation of somatic mutations in HCC

**Fig. 5:**
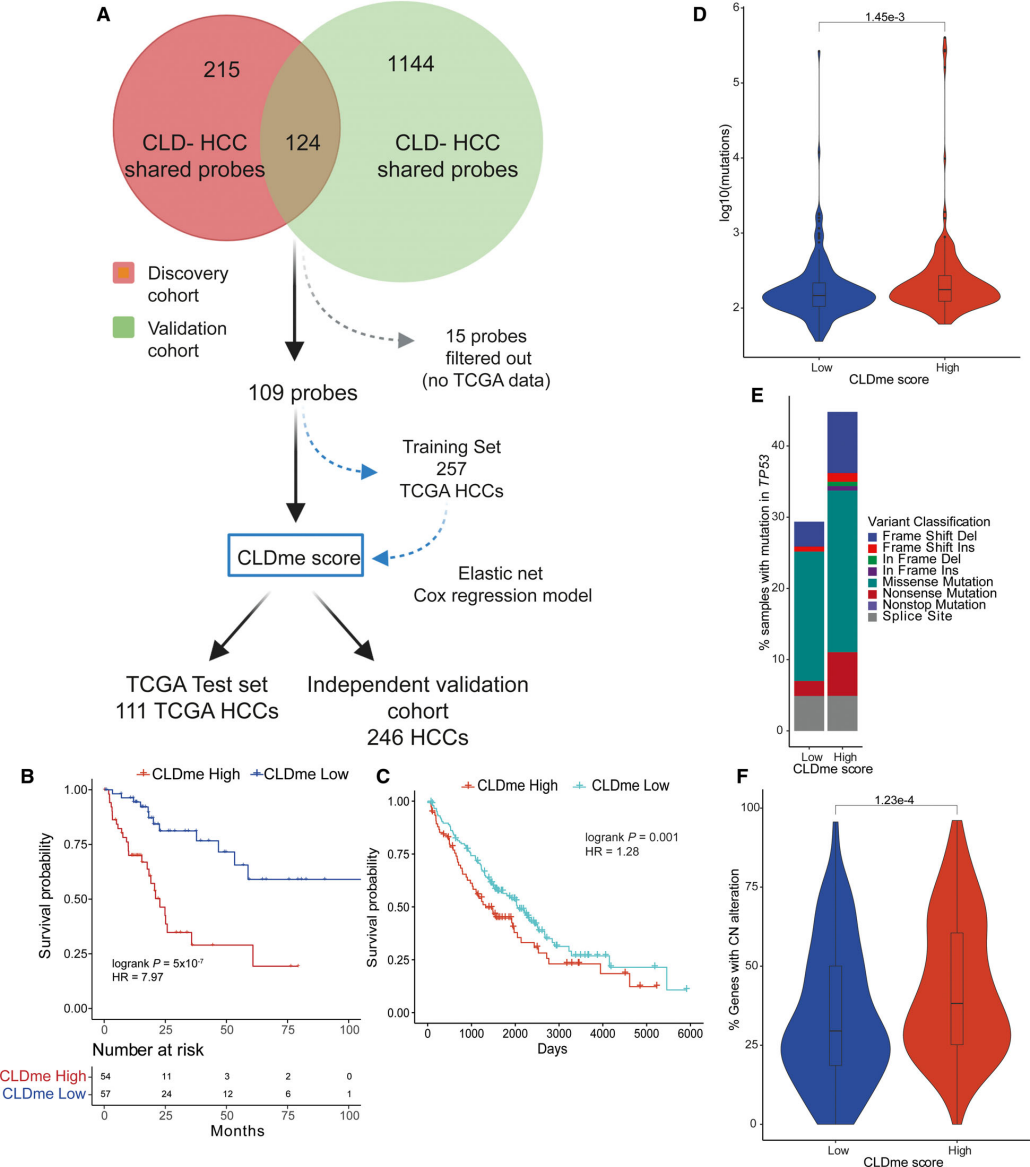
Implications for epigenetic priming in CLD on HCC. (A) The approach used to select CpG sites for elastic net regression using TCGA data. (B, C) Kaplan–Meier plots of survival probability in the TCGA test set (B, *n* = 111) and in an external validation cohort (C, *n* = 246), stratified into High/Low CLDme score. Logrank *P* value adjusted for disease stage and history. (D) Number of somatic mutations in TCGA samples stratified by CLDme score (163 high, 142 low). Violin plots show distributions of mutations per sample. Boxplot shows the mean and IQR. Whiskers show the range of the data up to 1.5 × IQR. Samples outside this range are plotted as points. *P* value computed from Wilcoxon’s test. (E) Barplot showing percentage of samples with *TP53* mutation types, stratified by CLDme (178 CLDme high, 183 CLDme low). (F) Extent of copy‐number alterations in TCGA samples stratified by CLDme score (178 CLDme high, 183 CLDme low). Violin plots, boxplots and statistics as for D. TCGA: The Cancer Genome Atlas; CLDme: chronic liver disease methylation; IQR: inter quartile range.

Next, we assessed whether the methylation state of the 124 DM CpG sites in both CLD and HCC samples in both data sets was of clinical relevance, using methylation, clinical and survival data of 368 HCCs from The Cancer Genome Atlas (TCGA) [39]. We randomly split the TCGA data set 70 : 30 into training (*n* = 257) and testing (*n* = 111) set, and after removing 15 CpG sites with missing values, we trained an elastic net regression model using the remaining 109 CpG sites to define a ‘CLD Methylation (CLDme)’ score for each sample (Methods, Fig. 5A and Table S1L). A multivariate Cox proportional hazards model, adjusted for disease history and stage (the only factors significantly associated with survival, Table S1M), showed a high CLDme score to be an independent predictor of poor survival in the test set of 111 TCGA samples (Fig. 5B, log‐rank *P* = 5e^−07^, HR = 7.97). We confirmed our findings using an independent data set of 241 patients [8] and showed that a high CLDme score was again significantly associated with survival independent of disease history and stage (log‐rank *P* = 0.001, HR = 1.28; Fig. 5C).

We next sought to determine whether the CLDme score was associated with genetic and transcriptomic alterations. Using the entire TCGA cohort, a differential expression analysis between CLDme high and low samples found 8 of 18 genes associated with DMRs in the initial discovery samples were DE between CLDme high and low samples (*q* < 0.05, deseq2 Wald test; Fig. S10A). On the genetic level, we found that the CLDme high samples had significantly more mutations than the CLDme low samples (*P* = 0.0015, Wilcoxon test; Fig. 5D). As mutations in *TP53* define a class of HCCs with poor prognosis, [60] we further asked whether CLDme was associated with *TP53* mutations. We found that *TP53* mutations were significantly enriched among CLDme high samples (44.5% vs 29% in CLDme Low, *P* = 0.0065, OR = 1.94, Fisher’s exact test, Fig. 5E). Similarly, we observed that CLDme high samples showed significantly higher copy‐number changes than CLDme low samples (*P* = 0.0001, Wilcoxon test; Fig. 5F).

Together, these data suggest epigenetic priming in CLD may have roles in HCC that go beyond a role in tumorigenesis. By shaping the transcriptional landscape of HCC and creating a more permissive environment for the acquisition of genetic alterations, aberrant methylation patterns in CLD may influence HCC outcome.

## 4 Discussion

In this proof‐of‐concept study, we demonstrate the extent of epigenetic and associated transcriptomic changes occurring in the progression from normal tissue, to CLD and HCC. We show that methylation changes acquired during CLD may not only have a role in tumorigenesis, but also sculpt the transcriptional landscape of the subsequent HCC, with implications for disease outcomes. We detected significant hypermethylation affecting genes previously reported to be aberrantly methylated and silenced, and incorporated in HCC prognostic scores, for example *RASSF1A*, *APC* and *P14* [8, 9, 61, 62, 63, 64]. However, here, using two cohorts, we expand upon these by showing the extent and impact of DNA methylation changes in CLD is more far‐reaching than has previously been reported, affecting genes for which aberrant methylation has not, to our knowledge, been reported in CLD, for example *CYP2C19*, *TSC2* and *TAGLN2*. Firstly, we showed that genes reported to be upregulated and, in some cases, to promote HCC progression, such as *HDAC11*, *UBD* (*FAT10*) and *TAGLN2* [57, 65, 66], are hypomethylated in CLD samples, suggesting these prognostically relevant epigenetic and transcriptional changes may arise before HCC has developed. Secondly, we showed that high CLDme score was associated with higher levels of *TP53* alterations, a poor prognostic indicator [60], suggesting epigenetic changes acquired during CLD may be permissive for genetic alterations with the potential to influence HCC prognosis. While derived from DNA methylation changes initially detected in a small dataset, we validated the prognostic relevance of our model in two independent cohorts of HCC patients.

Our results reflect the recently proposed ‘epigenetic priming’ model, whereby epigenetic changes induced by chronic exposure to cigarette smoke were shown to sensitise cells to an oncogenic *KRAS* mutation by promoting EMT in lung cancer, or the epigenomic alterations driven by obesity were detectable in precancerous colonic epithelium [7, 67]. Importantly, while many of the genes affected by epigenetic priming are not necessarily cancer drivers, in the case of hypomethylated/upregulated genes such as *UBD* and *CREB5*, these genes have been linked to prognosis and disease outcome [56, 68, 69]. We therefore hypothesise that epigenetic priming during CLD may have implications for HCC prognosis through two possible mechanisms: by sculpting the transcriptional landscape of the emergent HCC, and by creating a permissive environment for the acquisition of genetic alterations affecting genes such as *TP53* that influences outcome [70].

RNA‐seq analysis revealed the nature of transcriptional reprogramming during the progression from CLD to HCC. First, we observed increased expression of immune gene sets in the CLD samples but not the HCC samples. CLD is characterised by the continued expression of cytokines and recruitment of immune cells to the liver [71]. However, during progression to HCC there is a shift towards a suppressive immune environment allowing the growth of cancer cells [72]. Secondly, in keeping with the tissue regeneration and fibrogenic processes occurring during CLD [73], we found enrichment for genes associated with EMT in CLD samples. Beyond this, we also found gene sets, such as E2F and MYC targets, are upregulated in CLD and in HCC in a progressive manner. Indeed, the upregulation of E2F targets has been reported to define a subclass of HCC [74, 75]. Thus, tumorigenic transcriptional programmes may already be activated in CLD.

Our genome‐wide evaluation of epigenetic dysregulation in matched CLD and HCC revealed that some of the epigenetic alterations in HCC are already detectable in CLD and are associated with transcriptional dysregulation. Of note, we found that DM CpG sites in CLD more frequently affected CpG islands and shores than those in HCC, suggesting that the methylation alterations in CLD may have a greater effect on transcriptional regulation than those in HCC. We also hypothesise that metabolic perturbations on the transcriptional level, such as *MAT1A* loss, may contribute to the epigenetic reprogramming. *MAT1A* loss results in reduced SAM synthesis, which is a feature of both cirrhosis and HCC that leads to global hypomethylation in rat livers during hepatocarcinogenesis, and is associated with increased proliferation in human liver cancer cells [76, 77]. Our results support a model of epigenetic priming occurring in CLD prior to the development of HCC and, more interestingly, that the influence of epigenetic priming in CLD may go beyond a role in tumorigenesis as it has the potential to create a transcriptional environment that influences disease outcomes.

Expanding upon previous work on epigenetic changes in CLD and HCC [9, 61], here we show that methylation changes acquired during CLD associate with outcome and genetic alterations in HCC. Notably, we detected hypermethylation of CpG sites within the *O*‐6‐methylguanine‐DNA repair gene *MGMT*, concomitant with its downregulation in CLD and HCC. Loss of *MGMT* permits liver cancer development *in vivo*, but recent studies have variably found links and no link between *MGMT* methylation and HCC risk [78, 79, 80, 81]. As *MGMT* is the sole enzyme responsible for *O*‐6 methylguanine repair, its hypermethylation‐induced silencing, initiated during CLD, may result in increased rates of mutation. Indeed, loss of *MGMT* has been associated with *TP53* mutations in HCC [80]. Loss of *MGMT* expression, associated with methylation of its promoter, defines a subset of HCCs [80] and has been reported in tumour‐adjacent tissue from HCC patients; however, this loss of expression was without associated promoter hypermethylation as measured using methylation‐specific PCR [82]. In conjunction with our data showing the hypermethylation of nonpromoter CpGs in *MGMT*, which have been shown to correlate with *MGMT* expression in NAFLD, this may imply the loss of expression of *MGMT* in HCC may be initiated by non‐promoter methylation changes acquired during CLD, which become ‘locked‐in’ by promoter methylation, as has been reported in HCC [11, 80]. Future work will focus on defining whether *MGMT* loss is more associated with tumour emergence, or progression.

The effect of epigenetic changes on the genetic landscapes of HCC is further illustrated by the association between CLDme score and the overall tumour mutational burden and *TP53* mutations in HCC, suggesting that the epigenetic state when a driver gene mutation occurs may influence outcome. Indeed, despite the small cohorts used to discover CLD‐associated methylation changes, we showed that the prognostic relevance of the detected changes was consistent in two large‐scale cohorts. We also noted that the prognostic relevance of the CLDme score is not purely a result of altered levels of immune infiltration given the lack of association between CLDme score and the presence of lymphocytes or the ‘ImmuneScore’ as defined by xCell in the TCGA cohort (Fig. S10B–D). Between the two cohorts, we also observed that the difference in survival between CLDme high and CLDme low patients was less pronounced than in the TCGA. We posit that this discrepancy may be due to differences in the ethnicity of the patients included in the two cohorts. The TCGA is composed of 43% Asian patients, while the validation cohort was collected in Spain, France and the United States so is likely to have a lower proportion of Asian patients. Secondly, the validation cohort had median AFP levels of 51, whereas the TCGA had a median value of 15. Elevated AFP is associated with the CpG island methylator phenotype in HCC so this may also impact the methylomes of patients in the validation cohort, affecting the accuracy of prediction [70].

Other studies have shown the potential for methylation changes at specific gene promoters to predict hepatocarcinogenesis [9]. Therefore, an obvious extension of the work presented is to ask whether the CLD‐associated methylation signature may have predictive and prognostic potential. To test the feasibility of this, we performed the same array profiling on CLD tissue from six patients with decompensated liver disease and advanced CLD for > 10 years without HCC development. With this small cohort, we were able to detect a trend (*P* = 0.059) towards lower CLDme score in non‐progressing CLD, compared with HCC‐associated CLD (Fig. S11). While this remains to be validated in a larger cohort, these preliminary data indicate that a lack of this epigenetic dysregulation may be associated with a reduced risk of HCC emergence. While there was a strong inverse correlation between the methylation status of the identified DMRs and the expression of their associated genes, for genes such as *CYP2C19* and *TLDC2* the change in expression was disproportionate to the change in methylation. This observation may point to roles for other epigenetic mechanisms, such as altered patterns of histone modifications and chromatin organisation, in transcriptional regulation of these genes and in the progression of CLD to HCC. Indeed, ongoing research is focussed on the notion of the reversibility of changes to histone modifications occurring in the CLD‐HCC transition, and other groups have shown the susceptibility of epigenetic reprogramming (H3K27ac in particular) to therapeutic intervention to prevent the onset of HCC in mice [83].

## 5 Conclusions

In summary, we have shown that CLD and HCC samples from the same patient share broad transcriptional and epigenetic alterations, which are compounded in HCC. Our results highlight how methylation changes in CLD may help not only to create a transcriptional landscape favourable for HCC emergence, but that the influence of these changes may extend to consequences for disease outcomes. The development of the CLDme score demonstrates that epigenetic changes occurring in CLD and affecting both genes previously reported to be aberrantly methylated in CLD, as well as those we identify here, can be leveraged to predict HCC outcomes. Future studies will focus on identifying DNA methylation changes that may help identify CLD that would progress to HCC.

## Conflict of interest

AV has received consulting fees from Guidepoint, Fujifilm, Boehringer Ingelheim, FirstWord and MHLife Sciences; advisory board fees from Exact Sciences, Nucleix, Gilead and NGM Pharmaceuticals; and research support from Eisai. JML has received consulting fees from Eli Lilly, Bayer HealthCare Pharmaceuticals, Bristol‐Myers Squibb, Eisai Inc., Celsion Corporation, Merck, Ipsen, Genentech, Roche, Glycotest, Nucleix, Sirtex, Mina Alpha Ltd and AstraZeneca; and research support from Bayer HealthCare Pharmaceuticals, Eisai Inc., Bristol‐Myers Squibb, Boehringer Ingelheim and Ipsen.

## Author contributions

CKYN and SP conceived and supervised the study. JG performed the bioinformatic analysis. MC‐L, VP, SN, ST‐M and GB performed nucleic acid extraction, immunohistochemistry and sequencing reactions. CE and LMT performed histopathologic review of the samples. AV, JML M‐AM, TB, SP‐d‐P, SR‐T, MvF, SDS, OK and MHH provided the samples and the clinical information included in the discovery cohort and critically discussed the results. AV and JML provided the samples included in the validation cohort and critically discussed the results. JG, MC‐L, CKYN and SP interpreted the results and wrote the manuscript.

### Peer Review

The peer review history for this article is available at https://publons.com/publon/10.1002/1878‐0261.13154.

## Supporting information


**Fig. S1.** Schematic summarising the origin of samples used for methylation, transcriptomic, WES, and IHC analysis. WES: Whole exome sequencing; IHC: immunohistochemistry.
**Fig. S2.** Heatmap showing gene expression of top 500 most variably expressed genes across normal livers, CLDs and HCCs.
**Fig. S3.** Heatmap showing expression of genes differentially expressed in CLD, HCC or both, compared to normal livers.
**Fig. S4.** Genetic alterations detected in HCC are not present in matched CLD samples.
**Fig. S5.** DNA methylation in CLD, HCC, and normal liver.
**Fig. S6.** Venn diagram of CpG sites showing differential methylation in CLD and HCCs compared to normals.
**Fig. S7.** Overlap between methyl‐binding domain protein ChIP‐seq data and CLD‐HCC DMRs.
**Fig. S8.** Differentially methylated regions in CLD and HCC samples, compared to normal livers.
**Fig. S9.** Methylation changes in MGMT in CLD and HCC are conserved across cohorts.
**Fig. S10.** Characterisation of CLDme High and Low TCGA samples.
**Fig. S11.** CLDme scores in HCC, CLD, and nonprogressing CLD (NPC).

**Table S1.** (A) Cohort overview. (B) Differential gene expression ‐ CLD ‐ Normal. (C) Differential gene expression ‐ HCC ‐ Normal. (D) Genes DE in CLD and HCC compared to Normal. (E) Coding alternations in CLD and HCC pairs. (F) Copy number alterations in CLD and HCC pairs. (G) CpG sites DM in CLD compared to Normal. (H) CpG sites DM in HCC compared to Normal. (I) CpG sites DM in both CLD and HCC compared to Normal. (J) DMRs in both CLD and HCC compared to Normal, associated with DE genes. (K) CpG sites falling in DMRs from Table J. (L) Annotation of 109 probes used in prognostic model. (M) Univariate analysis of clinical features and survival in TCGA HCC cohort.

## Data Availability

The data that support the findings of this study are available on request from the corresponding author.
