# Supplementary material for: Epigenetic priming in chronic liver disease impacts the transcriptional and genetic landscapes of hepatocellular carcinoma
Source: Mol Oncol. 2021 Dec 29;16(3):665–82. doi: 10.1002/1878-0261.13154 (PMC8807355; doi:10.1002/1878-0261.13154)
Supplement: Supplementary file 1 — Fig. S1. Schematic summarising the origin of samples used for methylation, transcriptomic, WES, and IHC analysis. WES: Whole exome sequencing; IHC: immunohistochemistry. Fig. S2. Heatmap showing gene expression of top 500 most variably expressed genes across normal livers, CLDs and HCCs. Fig. S3. Heatmap showing expression of genes differentially expressed in CLD, HCC or both, compared to normal livers. Fig. S4. Genetic alterations detected in HCC are not present in matched CLD samples. Fig. S5. DNA methylation in CLD, HCC, and normal liver. Fig. S6. Venn diagram of CpG sites showing differential methylation in CLD and HCCs compared to normals. Fig. S7. Overlap between methyl‐binding domain protein ChIP‐seq data and CLD‐HCC DMRs. Fig. S8. Differentially methylated regions in CLD and HCC samples, compared to normal livers. Fig. S9. Methylation changes in MGMT in CLD and HCC are conserved across cohorts. Fig. S10. Characterisation of CLDme High and Low TCGA samples. Fig. S11. CLDme scores in HCC, CLD, and nonprogressing CLD (NPC). [file MOL2-16-665-s002.pdf]

Supplementary Figure Legends

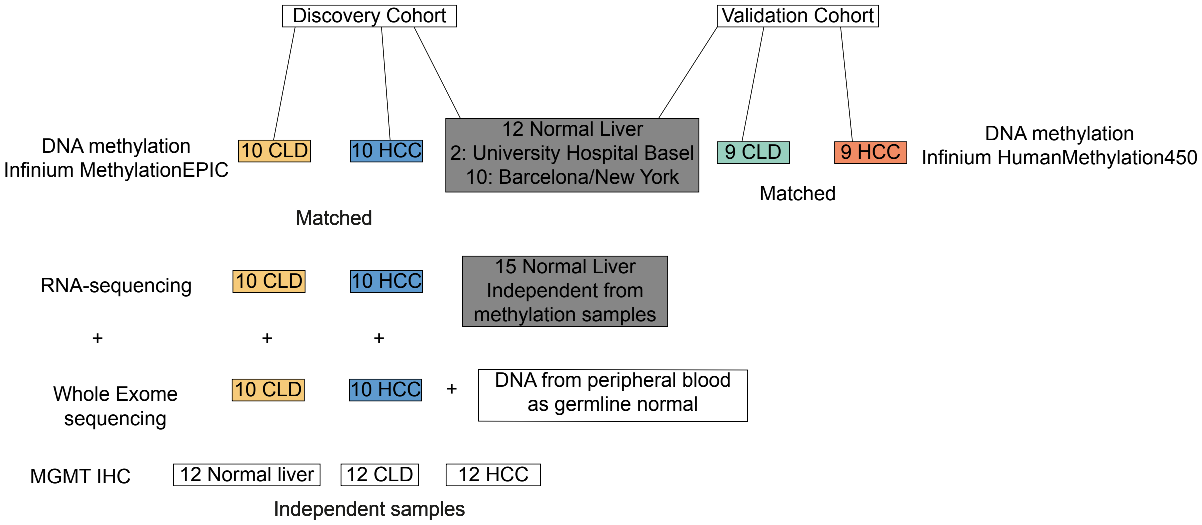

Fig. S1: Schematic summarising the origin of samples used for methylation, transcriptomic, WES, and IHC analysis.

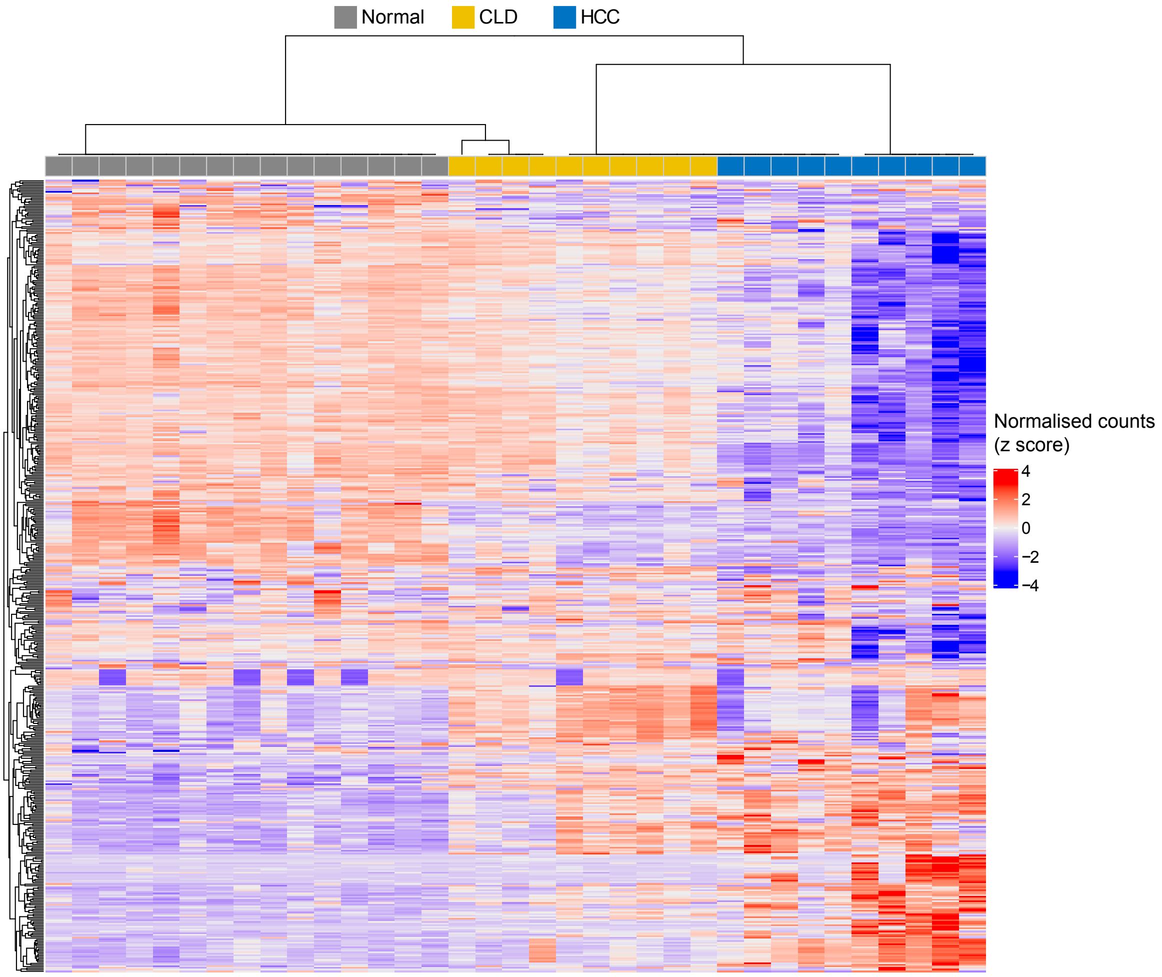

Fig. S2: Heatmap showing gene expression of top 500 most variably expressed genes across normal livers, CLDs and HCCs. Most variable genes were determined based on standard deviation across all samples. Dendrogram determined by consensus clustering. Each row is a gene, and heatmap colours show z scaled, normalised expression.

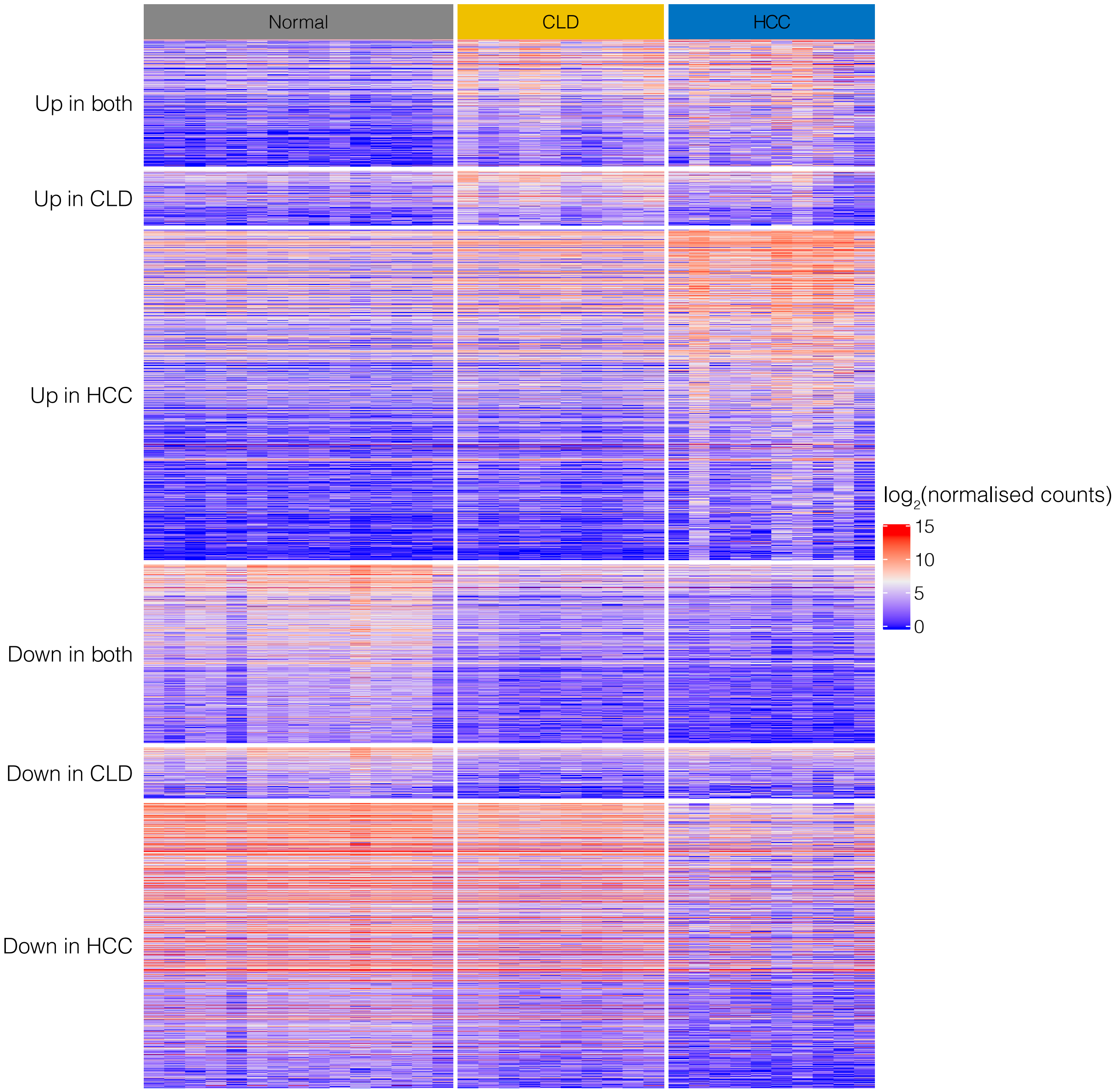

Fig. S3: Heatmap showing expression of genes differentially expressed in CLD, HCC or both, compared to normal livers. Each row is a gene, and heatmap colours show z scaled, normalised expression.

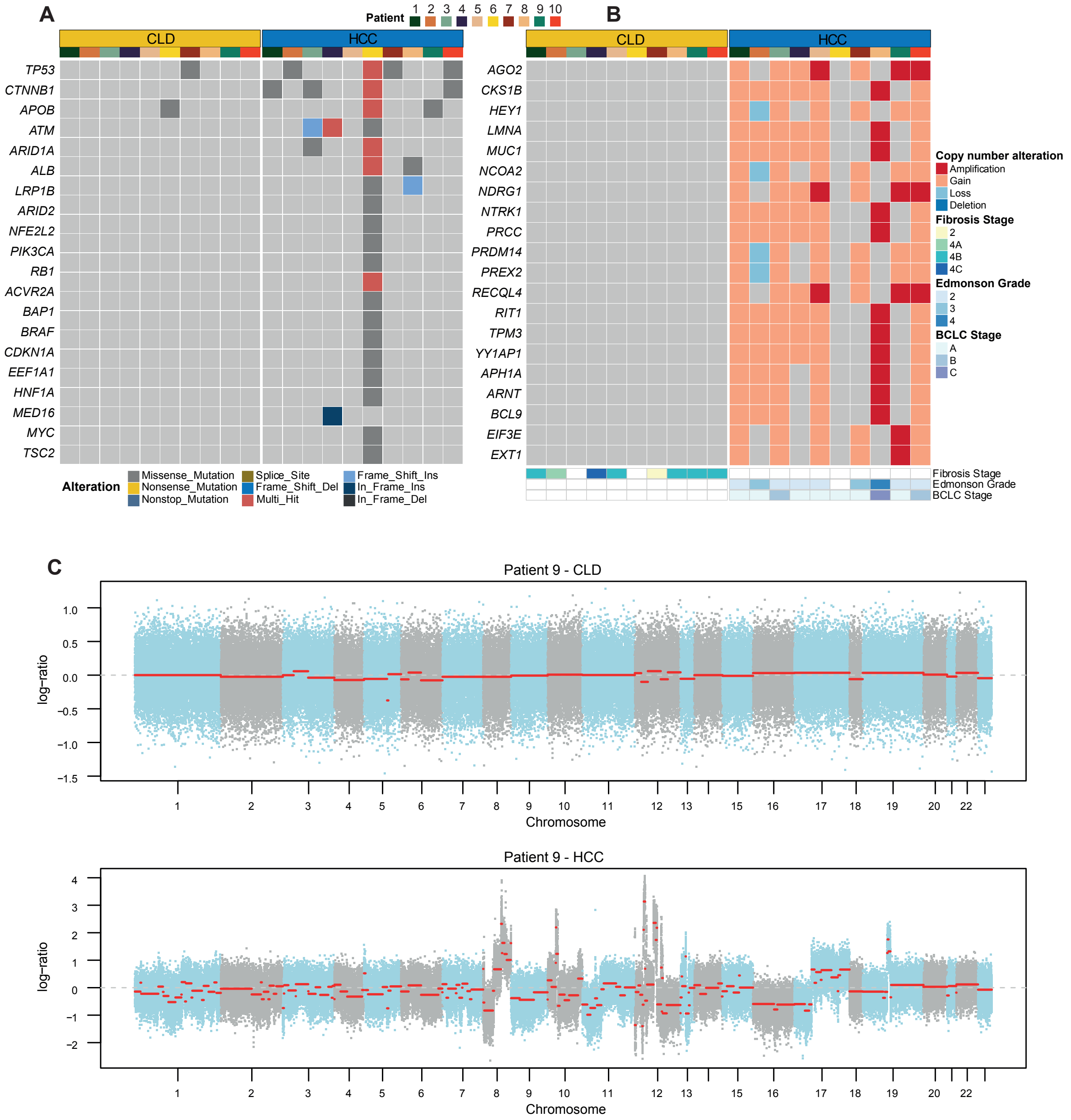

Fig. S4. Genetic alterations detected in HCC are not present in matched CLD samples. **A**) Summary of coding mutations in CLD and HCC samples from ten patients, affecting 20 frequently altered genes in HCC. The effects of the somatic alterations are color-coded according to the legend.

B) Summary of copy number alterations affecting 20 genes showing frequent CNA in HCC. Samples are annotated with fibrosis stage (CLD samples) and HCC samples with Edmondson and BCLC stage. **C**) Representative genome-wide copy number plot (Patient 9).

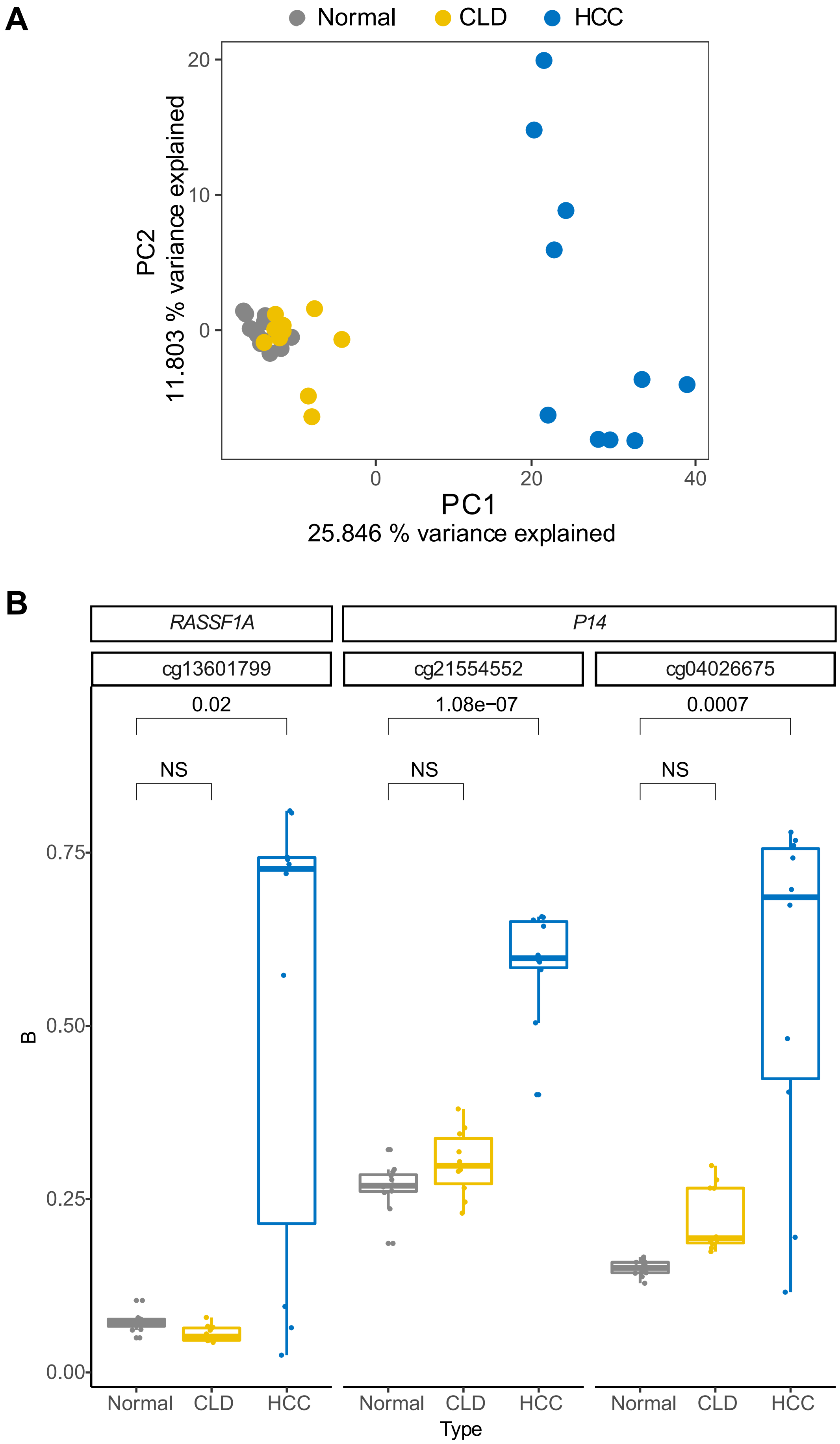

A
Normal
CLD
HCC
PC2
11.803 % variance explained
PC1
25.846 % variance explained
20
10
0
0
20
40
B
RASSF1A
P14
cg13601799
cg21554552
cg04026675
0.02
1.08e−07
0.0007
NS
NS
NS
B
0.75
0.50
0.25
0.00
Normal
CLD
HCC
Normal
CLD
HCC
Normal
CLD
HCC
Type

Fig. S5: DNA methylation in CLD, HCC, and normal liver**. A**) Normal liver and CLD samples are separated from HCC samples by PC1. **B**) Hypermethylation of CpG sites associated with *RASSF1A* and *P14* in HCC. B values for 12 normal livers and 10 paired CLD and HCC samples. *P* values from moderated t-test, limma.

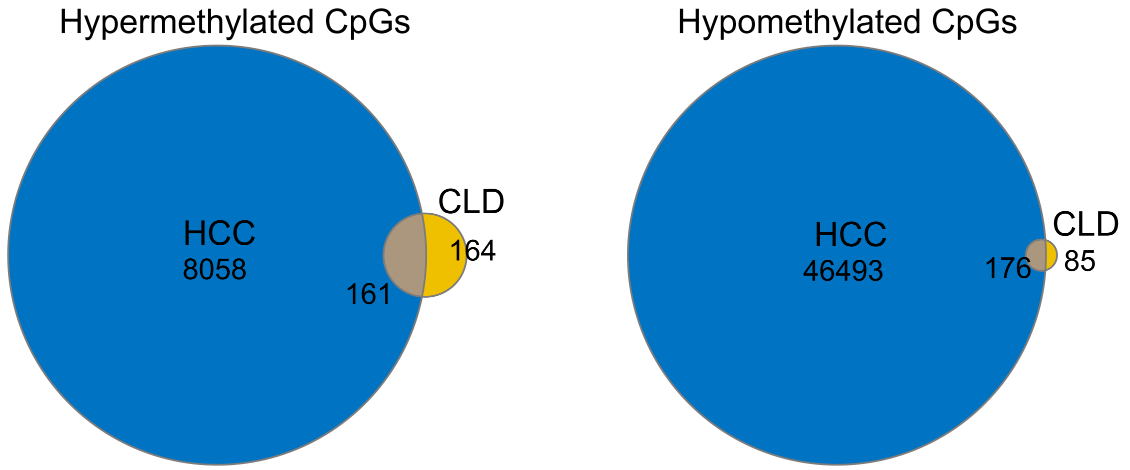

Fig. S6: Venn diagram of CpG sites showing differential methylation in CLD and HCCs compared to normals. Sites are split according to the direction of methylation change.

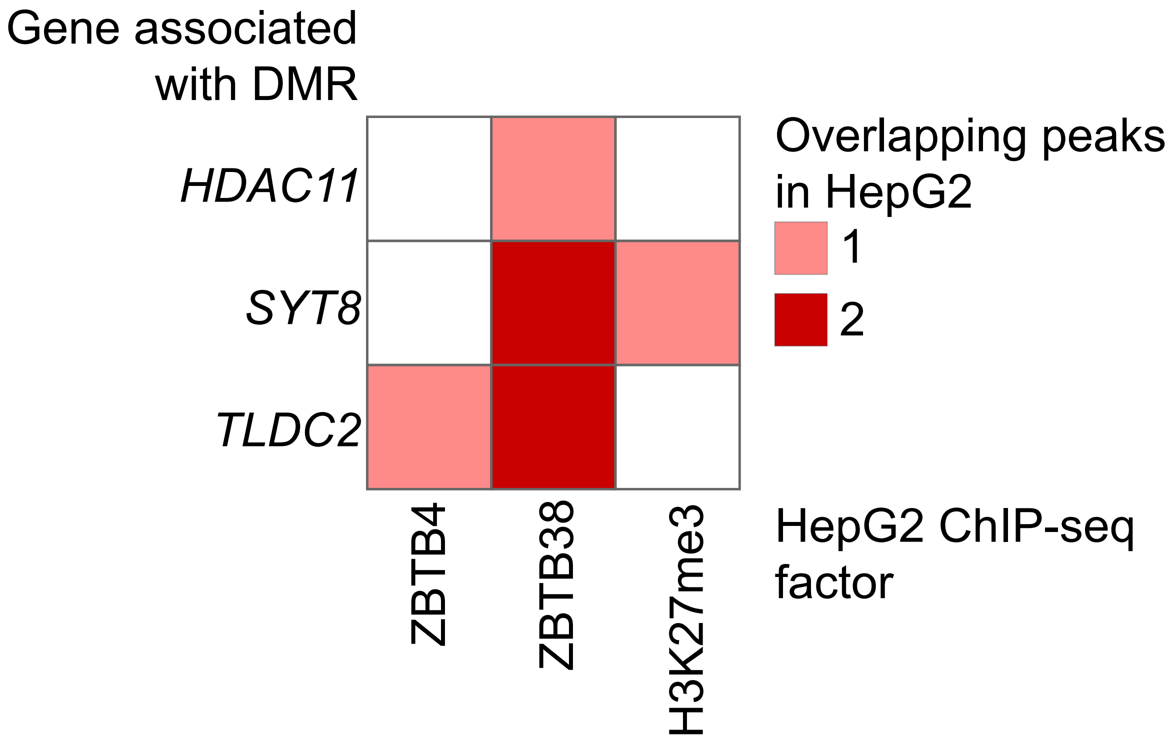

Fig. S7: Overlap between methyl-binding domain protein ChIP-seq data and CLD-HCC DMRs.

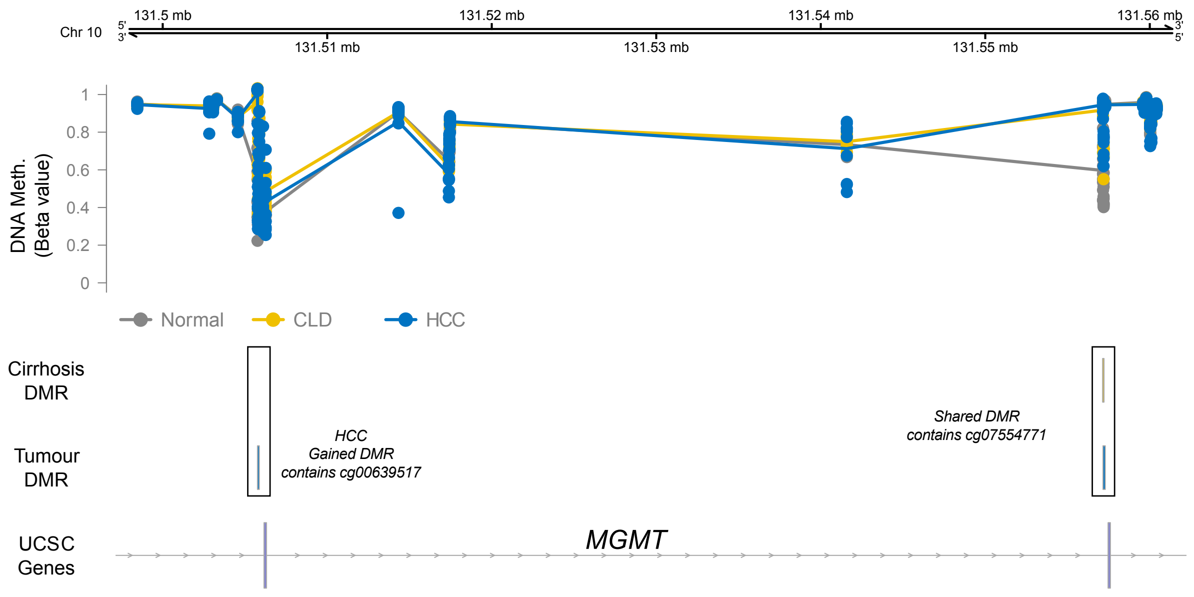

Fig. S8: Differentially methylated regions in CLD and HCC samples, compared to normal livers. Track plot of DMRs in *MGMT* shared between CLD and HCC and gained in HCC. DMRs were called by DMRcate with an FDR < 0.05 and change in B > |0.15|. CpG sites correlated with *MGMT* expression in Murphy *et al.* are highlighted.

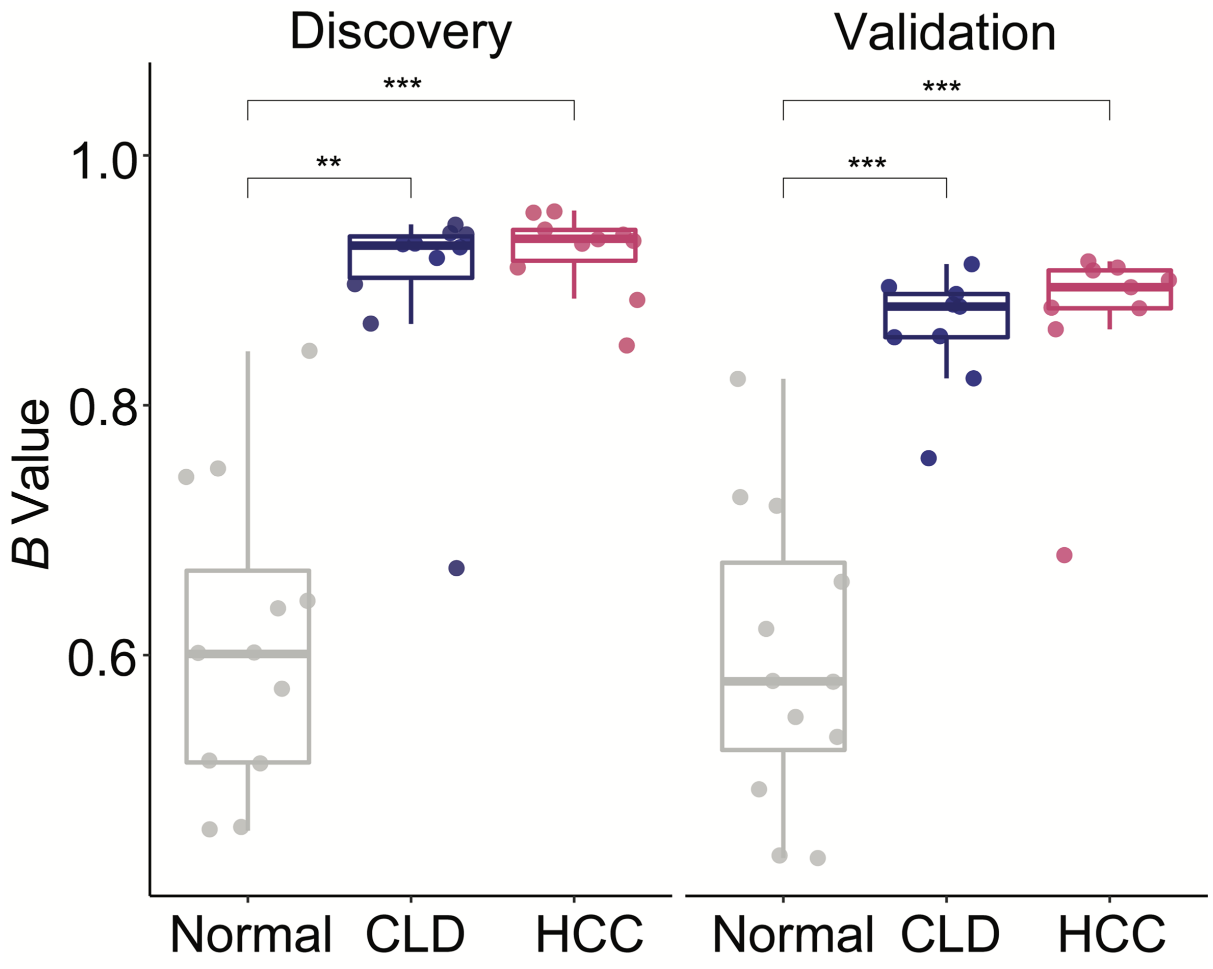

Fig. S9: Methylation changes in MGMT in CLD and HCC are conserved across cohorts. MGMT is hypermethylated in CLD and HCC compared to healthy livers. Methylation of cg07554771 in 12 healthy livers and 10 CLD and HCC pairs (discovery cohort) and a further 9 CLD - HCC pairs in the validation cohort. Wilcoxon Test.

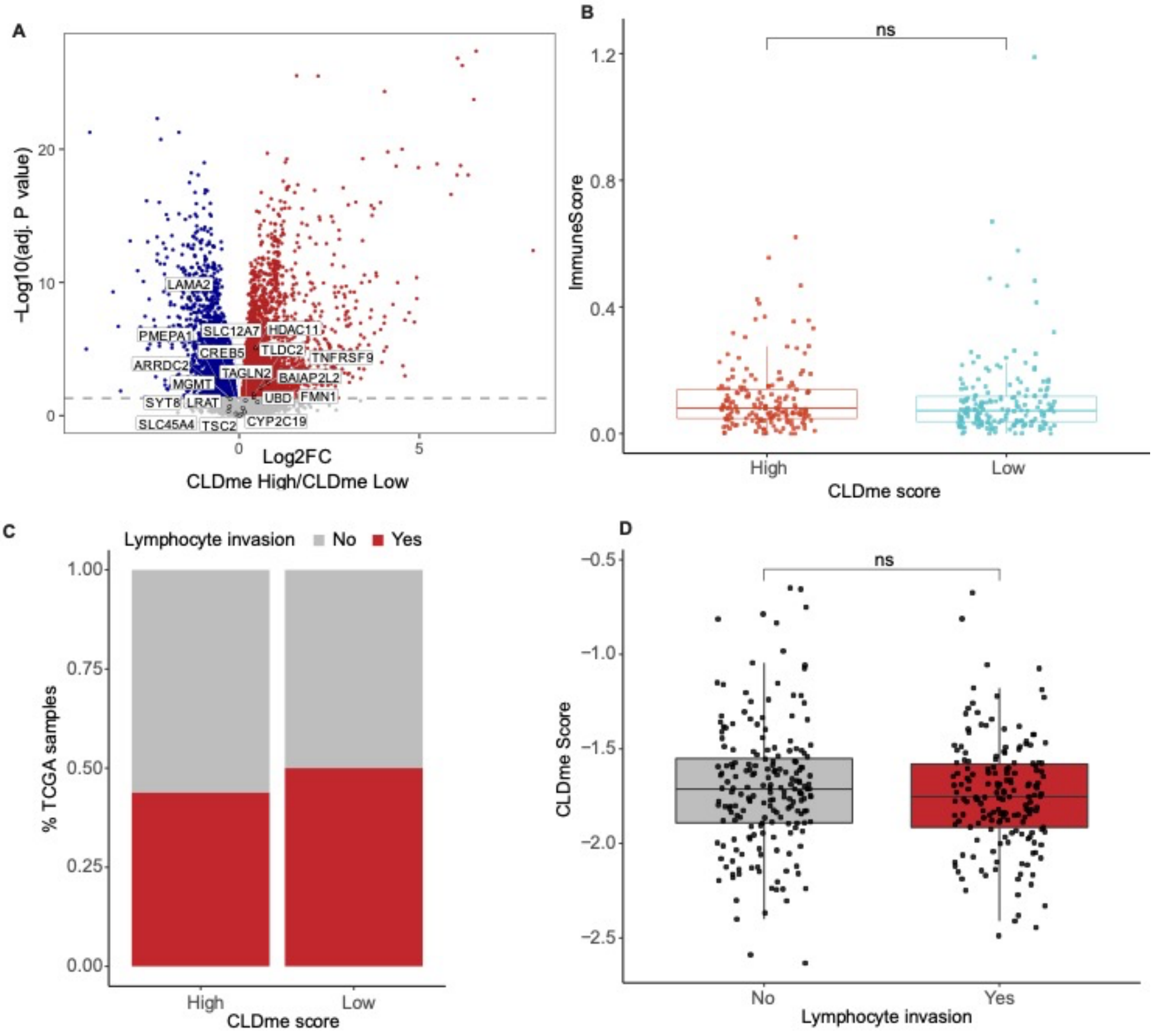

Fig. S10: Characterisation of CLDme High and Low TCGA samples. A) Volcano plot showing results of differential gene expression analysis comparing CLDme High to CLDme low tumours. 18 genes showing DE and associated with DMRs in the initial discovery samples are labelled. Dashed line shows significance threshold P < 0.05, DESeq2 Wald test. B) High and Low CLDme score TCGA samples show no difference in ImmuneScore. ImmuneScore was calculated for each sample as the sum of 11 cell types (‘B-cells', 'CD4+T-cells', 'CD8+ T-cells' , 'DC', 'Eosinophils', 'Macrophages', 'Monocytes', 'Mast cells', 'Neutrophils' and 'NK cells') calculated by xCell using the expression data for 360 TCGA samples. Wilcoxon test ns = non-significant. C) Percentage of TCGA samples, stratified by CLDme score, with and without lymphocyte invasion. D) CLDme score in TCGA samples with and without lymphocyte invasion. Wilcoxon test.

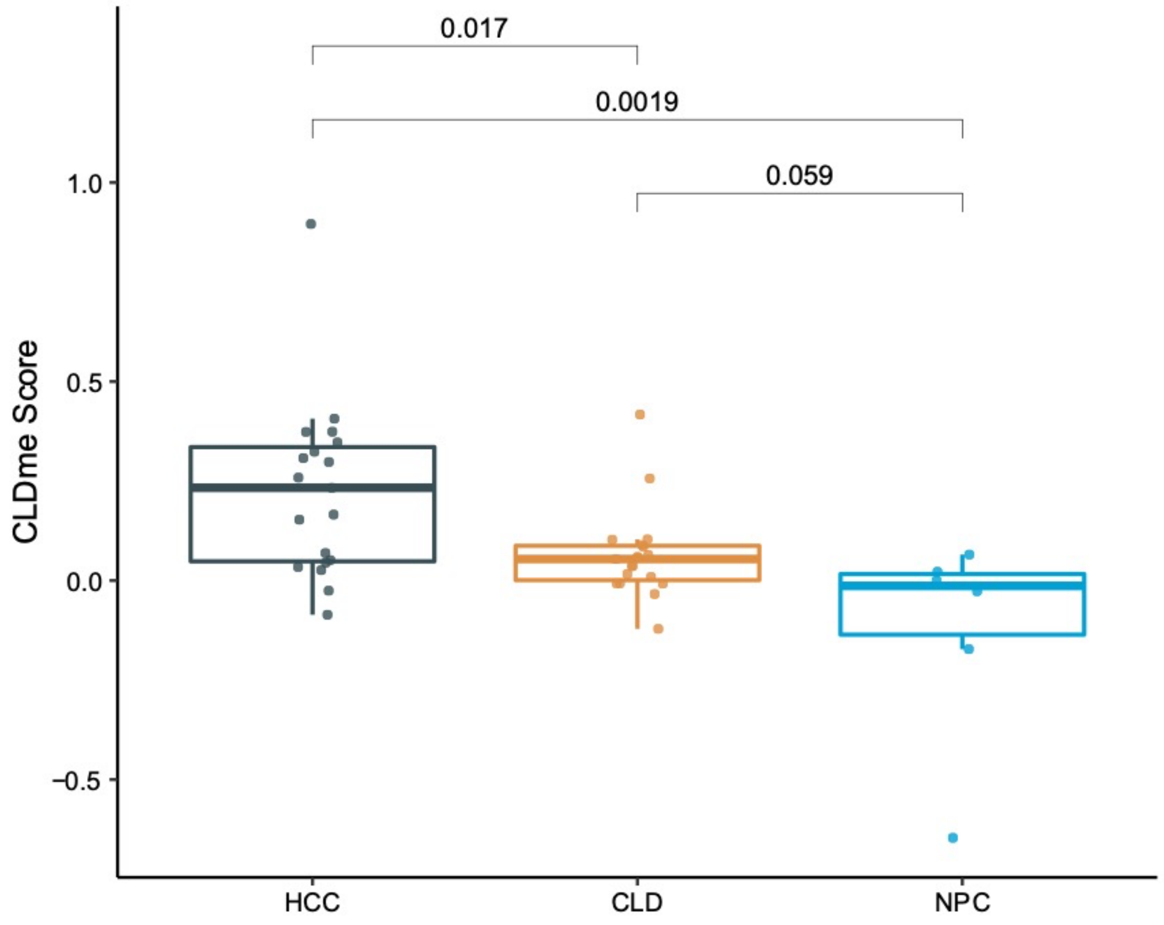

Fig. S11: CLDme scores in HCC, CLD, and non-progressing CLD (NPC). CLDme scores were generated for an additional 6 CLD patients with CLD for > 10 years, without HCC development, and compared with the CLD and HCC samples used throughout the study. Beta values were median centred before score generation to minimise cross-array batch effects. HCC n = 19, CLD n =19, NPC = 6. Wilcoxon Test.
